# Chunking and Redintegration in Verbal Short-Term Memory

**DOI:** 10.1037/xlm0000762

**Published:** 2019-09-30

**Authors:** Dennis Norris, Kristjan Kalm, Jane Hall

**Affiliations:** 1 Medical Research Council Cognition and Brain Sciences Unit, University of Cambridge

**Keywords:** memory, STM, chunking, computational modeling

## Abstract

Memory for verbal material improves when words form familiar chunks. But how does the improvement due to chunking come about? Two possible explanations are that the input might be actively recoded into chunks, each of which takes up less memory capacity than items not forming part of a chunk (a form of data compression), or that chunking is based on redintegration. If chunking is achieved by redintegration, representations of chunks exist only in long-term memory (LTM) and help to reconstructing degraded traces in short-term memory (STM). In 6 experiments using 2-alternative forced choice recognition and immediate serial recall we find that when chunks are small (2 words) they display a pattern suggestive of redintegration, whereas larger chunks (3 words), show a pattern consistent with data compression. This concurs with previous data showing that there is a cost involved in recoding material into chunks in STM. With smaller chunks this cost seems to outweigh the benefits of recoding words into chunks.

Memory for lists of words, digits, or letters tends to improve when the input can be organized into familiar chunks (e.g., Cowan, Chen, & Rouder, 2004; Miller, 1956; Simon, 1974). Miller argued this was because the chunk was the underlying unit of storage in short-term memory (STM). The capacity of STM was not determined by the number of items that could be stored, nor by the amount of information that could be stored, but by the number of chunks. Famously, Miller suggested that the capacity of STM was seven plus or minus two chunks. Later work, notably by Cowan (2001), has favored a lower estimate of capacity of three to five chunks. However, the notion that the underlying capacity of STM is determined by the number of chunks remains the same. Miller suggested that the simplest way to perform chunking was “to group the input events, apply a new name to the group, and then remember the new name rather than the original input events” (p. 93). That is, chunking is achieved by recoding the input into a different vocabulary.

Chunking by recoding can be seen as an example of data compression. This capitalizes on redundancy in the input to form a new representation of the input or message that can be transmitted using fewer bits of information (for an extensive discussion of data compression in STM, see Norris & Kalm, 2019). Although there is no doubt that the presence of familiar groups of items in the input leads to better performance, until recently there has been little direct evidence that this comes about through data compression (Brady, Konkle, & Alvarez, 2009; Thalmann, Souza, & Oberauer, 2019). Indeed, in the case of verbal STM there is evidence for the operation of a quite different mechanism—redintegration (Botvinick, 2005; Botvinick & Bylsma, 2005; Jones & Farrell, 2018). According to a redintegration view, chunks are not recoded into a different representation that is then stored in STM. Instead, the representations of chunks exist only in LTM, and these LTM representations allow degraded traces in STM to be more readily reconstructed. The benefit of having chunks is thus not mediated by recoding and hence does not lead to compression of the representations in STM. Although the basic prediction from both accounts is that performance is improved by the presence of chunks, there are circumstances where they make different predictions. Here we report six experiments designed to compare these predictions.

The paradigmatic example of chunking by recoding comes from a study by Smith, reported in Miller (1956). Smith taught himself to recode lists of binary digits as octal digits (e.g., recode 101 as 5). Every chunk of three binary digits could therefore be recoded as a single octal digit. Smith could remember about 12 octal digits, but by recoding triples of binary digits as octal he could recall about 36 binary digits. Cowan and colleagues (Chen & Cowan, 2005, 2009; Cowan & Chen, 2008; Cowan et al., 2004; Cowan, Rouder, Blume, & Saults, 2012) have performed more systematic experimental investigations of chunking to determine whether the capacity of verbal STM really is determined by the number of chunks that can be stored. The general procedure in these studies was to have participants learn multiword chunks in a cued recall task. They were then tested on either free recall, serial recall, or forced-choice recognition of lists containing different numbers of chunks. Memory performance typically improved when lists were made up of larger chunks.

In one study, Chen and Cowan (2009) trained participants with either pairs of words (e.g., *brick–hat, king–desk*) or individual words (singletons), and then had them perform serial recall with lists of two, four, six, eight, or 12 singletons, or four or six learned pairs. Participants had to perform articulatory suppression during list presentation and then to type their response. Responses were scored both in terms of the number of items recalled in their correct position (strict scoring) or the number of items recalled anywhere (lenient scoring). If recall were determined by the number of chunks that could be stored, performance on lists of four pairs should be the same as lists of four singletons because they both contain the same number of chunks. That is, twice as many items should be recalled when lists comprise of pairs than when they comprise of singletons. This is what was found, but only when using lenient scoring. With strict scoring, the number of items recalled was also influenced by the number of items in the list. In an earlier study, Chen and Cowan (2005) had found no sign of a chunk-based limit when participants were not required to perform articulatory suppression. In fact, for eight-item lists there was no difference between lists consisting of either eight singles or four pairs. Considering both of these studies together, Chen and Cowan (2009) concluded that, “There is no apparent chunk-based constant capacity for strict serial recall” (p. 1425).

Cowan et al. (2012) extended this work by examining recall of a much larger range of list lengths and chunk sizes. As in their previous studies, they found that recall performance was not a simple function of the number of chunks in the list. Recall tended to decrease as the number of items in the list increased, even when the number of chunks remained the same. When lists were made longer by adding more singletons, performance was better than would be expected by constant chunk capacity. Cowan et al. fitted a number of different mathematical models of chunking to the data. The model that provided the best fit to the data (Model VIII) added two parameters to the simple constant capacity model. The first allowed for the possibility of chunk decomposition; some chunks may break down into their components and no longer function as a single chunk. This accounts for the poorer than expected performance with larger chunks. The second allowed for the possibility that some chunks may be stored in activated LTM, which has unlimited capacity. This accommodates the finding that recall does not decline as much as would be expected when lists contain more singletons. Note that none of these models had anything to say about how chunks are represented in either STM or LTM.

Although the extra factors in Cowan et al.’s (2012) model mean that performance is not a direct function of the number of chunks in the input, the model nevertheless retains the assumption that the underlying capacity of STM is determined by the number of chunks that can be stored. If there are C chunks and S slots, each chunk will be stored in STM with a probability S/C. Cowan et al.’s mathematical models have nothing to say about the form that chunks might take in STM. Earlier, Chen and Cowan (2009) suggested that “An advantage of acquiring multiword chunks is that it is then unnecessary to keep each word in capacity-limited working memory, but just some index to each chunk, perhaps the chunk’s first word” (p. 1428). This represents the simplest possible account of chunking. Chunking need not require chunks to be recoded into a completely different vocabulary (e.g., binary into octal) but might simply involve deleting redundant information.

## Evidence for Compression

Although chunking can be seen as a form of data compression, few studies of STM (Brady et al., 2009; Chekaf, Cowan, & Mathy, 2016; Huang & Awh, 2018; Jones & Farrell, 2018; Mathy & Feldman, 2012; Thalmann et al., 2019) have explicitly considered chunking in this way. Mathy and Feldman proposed that serial recall from STM may involve data compression. They found that participants had better memory for lists of digits when they contained runs of increasing or decreasing digits. They suggested that the presence of regular patterns enabled the lists to be compressed so as to take up less capacity, but they did not offer any suggestions as to how that compression might operate.

One of the first indications of a genuine effect of data compression in memory came from a study of visual STM by Brady et al. (2009). In their first experiment participants saw a display consisting of four circles arranged in a diamond. Each circle had two concentric rings of different colors, and the displays always contained eight different colors. The display was presented for 1 s after which they saw a second display that indicated which ring they had to recall. Brady et al. varied the frequency with which different colors were paired together in the circles. Individual colors always appeared with equal frequency. Not surprisingly, colors appearing in the pairs that had been presented more frequently were recalled better. Furthermore, their estimate of the number of items that could be held in memory increased over the course of the experiment. However, the critical finding was that, in displays containing high probability pairs, recall of colors in low probability pairs also improved relative to displays containing only low probability pairs. This implies that the presence of high probability pairs allows the representation of the entire display to be encoded more efficiently. The data can be interpreted in terms of chunking on the assumption that high probability pairs come to be treated as chunks, which take up less memory capacity and hence free up space for pairs that do not form chunks. Thus, chunking leads to data compression.

Brady et al. (2009) constructed a Bayesian model of how their participants learned the probabilities of pairs. Treating each pair as a separate item (i.e., blue outside, red inside might be pair *x*) they showed that performance was inversely correlated with the length of the Huffman code for the display. A Huffman code is a compression algorithm that assigns shorter representations to more frequently occurring symbols in the input. Even though memory capacity as measured in terms of number of items stored increased over the course of the experiment, the estimated capacity expressed in bits, as derived from the effective Huffman code, remained constant. That is, there was no evidence that practice with the task improved underlying memory capacity, but it does improve how that capacity can be used. Although Brady et al. determined memory capacity in terms of the length of a Huffman code, they are neutral with respect to whether compression is performed by something akin to Huffman coding or chunking. Indeed, they show that a chunking model can also give a good account of their data. They suggest that chunking can be thought of as a discrete approximation to a more graded form of compression. However, as they note, the standard Huffman coding algorithm is probably a poor model of the psychological processes involved. Huffman coding is ill-suited to modeling the process of adapting to changes in probabilities over time.^1^ A Huffman code would need to be continually recomputed over all possible color pairs and the codes assigned to different pairs will change as their probabilities change. In effect, the label assigned to a particular chunk would keep on changing throughout the course of the experiment. If some items come to be presented more often, this naturally changes the relative probabilities of all items and the codes for all items will need to be updated.

Brady et al.’s (2009) data provide an important qualification to Miller’s claim that, “The span of immediate memory seems to be almost independent of the number of bits per chunk” (p. 93). In Brady et al.’s experiments there is an improvement in performance due to chunking, but the memory capacity in bits remains constant. Chunking has enabled that capacity to be used more efficiently in coding the choices that must be made in the experiment.

Thalmann et al. (2019) investigated compression in verbal STM using similar logic to the Brady et al. (2009) study. As in Cowan et al.’s (2012) experiments, they familiarized participants with three word chunks, or used existing three-letter acronyms. They found that recall of novel three-word triples was improved when the list also contained a familiar chunk. That is, as in Brady et al.’s study, it appeared that the presence of a chunk in the list made more storage capacity available for other items in the list. The experiments to be reported here use a similar design, however, in addition to investigating the idea that chunking is achieved by some form of data compression, we also address the possibility that chunking may sometimes be entirely a consequence of redintegration.

## Redintegration and Bayesian Inference

A distinctive feature of chunking by data compression is that chunking must change the nature of the representations stored in STM. To gain the benefit of compression the input must be recoded into a different form than the original input. An alternative possibility is that chunks are represented only in LTM and the contents of STM remain unchanged. According to this view, whenever information to be remembered contains familiar chunks, memory will improve as a consequence of redintegration (Botvinick, 2005; Botvinick & Bylsma, 2005; Brown & Hulme, 1995; Hulme, Maughan, & Brown, 1991; Hulme et al., 1997; Jones & Farrell, 2018; Lewandowsky & Farrell, 2000; Poirier & Saint-Aubin, 1996; Roodenrys & Miller, 2008; Schweickert, 1993; Thorn, Gathercole, & Frankish, 2002). That is, the presence of LTM representations of chunks facilitates reconstruction of degraded traces in STM. Redintegration has been proposed, for example, as an explanation for the superior recall of high- than low-frequency words, or words than nonwords (Hulme et al., 1991; Hulme, Roodenrys, Brown, & Mercer, 1995). Redintegration can be characterized as a process of Bayesian inference (Botvinick, 2005; Botvinick & Bylsma, 2005); chunks in LTM provide a set of priors which can be used to compute the posterior probabilities of list items given the data in memory. According to Bayes’s theorem,

$$posterior_{i}\propto likelihood_{i}\cdot prior_{i}.$$

With a more degraded representation in STM, representations with stronger priors (sequences of words forming familiar chunks in LTM) will necessarily be better recalled than representations with weaker priors (no chunks in STM). Chunks, in effect, create a bias toward recalling more probable items. Consistent with this, Hulme et al. (1997) reported that low-frequency words tended to be recalled as a similar sounding high-frequency word (“list” substituted for “lisp”). Consider the case where the item to be remembered is “lisp” but the representation of “lisp” in STM is degraded. If there is some ambiguity in the representation there will be some likelihood that the input is actually “list.”

However, even if *likelihood*_lisp_ > *likelihood*_list_, if *prior*_list_ is much greater than *prior*_lisp_, *posterior*_list_ may well be greater than *posterior*_lisp_. In contrast, given a degraded representation of “lisp,” the likelihood of (evidence for) a dissimilar word such as “truth” will be very small, and even a large frequency imbalance in favor of “truth” will not lead to it being recalled in error.

Botvinick and Bylsma (2005) found evidence for a similar phenomenon in serial recall which they called the “good-neighbor” effect. They tested recall of sequences of pseudowords after participants had been exposed to an artificial grammar. In the exposure phases, participants might hear sequences including ABABAB, ABBAAB, and AABABB, where ABABAB occurs more frequently than the other two. The sequence ABBAAB therefore has a higher probability “good neighbor” ABABAB, which differs from it by just one transposition (BA → AB). In contrast, the sequence AABABB, which has the same probability of occurring in the experiment, does not have a good neighbor—it cannot be transformed into any other sequence in the experiment simply by making a single transposition. Following exactly the same logic as for the Hulme et al. (1997) study, a degraded representation of ABBAAB will sometimes be erroneously recalled as its higher frequency good neighbor—ABABAB. It will therefore tend to have a higher error rate than a sequence such as AABABB which does not have a good neighbor. This is what they found. Although this is exactly what would be expected from a Bayesian account of redintegration, there is no reason to expect such a result if people had learned sequences by chunking.

## Distinguishing Between Compression and Redintegration

Given that both data compression and redintegration predict that lists of items containing familiar chunks will be easier to remember than those that do not, is it possible to distinguish between them? Fortunately, as demonstrated by Brady et al. (2009) and by Thalmann et al. (2019), there are some conditions where they make different predictions.

For expository purposes, we will frame our discussion of compression in terms of a simple fixed-capacity slot model where each slot can store a single chunk. A chunk might be a single word or some representation of (or pointer to) a multiword chunk. Compression is achieved by recoding single words into larger chunks. However, the logic of the argument applies quite generally to all forms of compression. For example, Brady et al.’s (2009) model makes no commitment to slots.

Consider the task of recalling a list where only some of the items form chunks. The compression view implies that the formation of chunks will release capacity that can be used to store more items. For example, if chunking two words together frees up an extra slot in memory, that slot can be used to store one more single item or one more chunk. That is, chunking should benefit all items in the list, not just the multiword chunks themselves. This will also be the case for Cowan et al.’s (2012) model where the probability of recalling a chunk is a function of the ratio of the number of slots to the number of chunks.

A further prediction of the simple model is that the presence of chunks should benefit multiword chunks and singletons to the same extent. The probability of storing each chunk is the same as the probability of storing each singleton; chunks and singletons have the same probability of occupying one of the slots. Given that the probability of remembering an item in multiword chunk must be the same as the probability of remembering the chunk, the probability of recalling any single word will be the same regardless of whether or not it is part of a chunk. The chunking model therefore predicts that chunking will improve the recall of all items in the list, and they will all be improved to the same extent.^2^

In contrast, the redintegration view predicts that chunking should have no influence on the amount of information or on the form of the representations actually stored in STM. Chunking does not influence the contents of STM, merely how well they can be retrieved. Consequently, redintegration will benefit only those items that form chunks. Overall performance will improve as the number of chunks in a list increases, but this should be entirely due to superior recall of the chunks. The performance on the remaining items should remain constant.

The critical difference between the two accounts is therefore that compression assumes that chunking alters the representations in STM. The formation of chunks in STM frees up STM capacity, which can then be used to store more items. In redintegration theories, chunks exist only in LTM. A multiword chunk is represented in STM in exactly the same way as that sequence of words would be if they did not form chunks. Redintegration benefits only those items that correspond to chunks in LTM and will not help other items in a list.

In the following experiments we test the predictions of these accounts using the same procedures as Cowan et al. (2012). We vary the size of the chunks used in our different experiments (two words in Experiments 1–3, three in Experiments 4–6) to see whether this has an influence on whether chunking is achieved by compression or redintegration. In the first three experiments, the chunks are prelearned pairs of words and in the next three experiments the chunks are word triples. Our procedure differs substantially from Thalmann et al. in that we used the same immediate serial recall and 2AFC recognition tasks used by Cowan. Thalmann et al. used a nonstandard cued serial recall procedure. In their experiments, words or letters were presented one at a time from left to right on an imaginary 3 × 3 grid. Each row corresponded to a learned chunk, three items in a novel sequence, or a single item. At recall, participants were cued to recall the items from each row in the correct order with the order of the cued rows being unpredictable. By requiring participants to recall each row, which might be a whole chunk, separately, they may have encouraged use a chunking strategy in a way not representative of standard whole list recall methods. Participants are forced to prepare to recall the whole chunk as a unit. It remains possible that their chunking effects might be due to chunks being easier to manipulate in this task rather than to any intrinsic advantage in STM for chunks. Furthermore, they used only this variant of serial recall.

## Experiments

Whereas Cowan et al. (2012) focused on performance for the entire experimental lists and how that varied as a function of list length in items and chunks, here our concern will be with lists containing a fixed number of items but where the number of chunks is varied. For example, in Experiments 1–3, lists were seven-items long and could either contain no prelearned pairs (only singletons) or one, two or three pairs plus singletons. Following Cowan and colleagues we refer to singles, pairs and triples as each being a single chunk. Each should occupy one slot in memory. Consequently, lists with more pairs or triples are described as containing fewer chunks. Experiments 4, 5 and 6 use lists where the chunks are triples. The central question here is what happens when more of the items in a list form chunks? Will that confer an advantage to all items in the list or only to items in those chunks? That is, will we see evidence of compression or will we see evidence of redintegration?

Experiments 1, 2, and 4 use the same forced-choice recognition task used by Cowan et al. (2012), whereas Experiments 3, 5, and 6 use an immediate serial recall task modeled on the experiments of Chen and Cowan (2005, 2009). Experiment 5 used six-item lists and the remainder used seven-item lists.

Although Chen and Cowan (2005, 2009) found that recall benefitted from chunking in all of their studies, their effect of chunking was greatest when lists were presented under articulatory suppression. Only then did they find any evidence of constant chunk-based capacity. Even that was only observed with their lenient scoring method (number of items recalled regardless of position). Therefore, to maximize the benefit of chunking in our experiments we had our participants perform suppression in all but Experiment 6. Note that Cowan et al. (2012) used articulatory suppression in all of their experiments. All experiments were approved by the Cambridge Psychology Research Ethics Committee (CPREC 2009.57). In all six experiments we used 28 participants, which is more than used by Thalmann et al. (2019) in their first two experiments (*n* = 20) and similar to the number used by Cowan et al. (2012) in their three experiments (*n*s = 26, 26, and 27). Furthermore, Experiment 2 is a near-replication of Experiment 1. The design of all six experiments is shown in Table 1. All of our analyses are based on Bayes factors, and the results of all six experiments show that we have sufficient power to both reject and accept the hypotheses of interest.[Table-anchor tbl1]

### Experiments 1–3: Two-Item Chunks

#### Experiment 1: 2AFC recognition

In the experiments reported by Cowan and colleagues, their general procedure was to vary the length of the lists while maintaining the number of chunks. In our experiments the list length was fixed but the number of chunks within the list varied. All lists contained seven words, and lists could contain zero, one, two, or three two-word chunks, with the remaining words being singletons that had never been learned as part of chunks. The order of the pairs and singletons was randomized. This mixing of pairs and singletons inevitably imposes constraints on the location of the singletons. In lists containing only one singleton, that item can never appear in Positions 2, 4 or 6. In all three experiments, there were eight blocks of eight trials. Apart from the differences in list composition, the general procedure was modeled as closely as possible on that used by Cowan et al. (2012).

In Experiment 1 we presented lists in which number of words was always seven, but number of chunks varied as shown in Table 2.[Table-anchor tbl2]

All experiments were approved by the Cambridge Psychology Research Ethics Committee and participants gave written consent to take part in the study. Participants were given a verbal debriefing when requested. All experiments lasted approximately 75 min and participants were paid 10UKP for participation.

##### Participants

Twenty-eight members of the Cognition and Brain Sciences Unit Volunteer Panel completed the experiment (20 women, *M*_age_ = 21.4 years).

##### Materials and procedure

The experiment consisted of three phases: chunk familiarization, articulatory suppression training, and list memory. The words were presented on a computer screen in an Arial 12-point lowercase typeface using DMDX (Forster & Forster, 2003), All stimuli were constructed from a pool of 44 word pairs (see the Appendix). Most of the word pairs are likely to have already been familiar (e.g., grass seed, oil can, radio show). For each participant, 12 pairs were selected at random to be used as complete pairs. For each participant either the first or the second words of the remaining 32 pairs were used as the singletons. Stimulus sequences were created by programs written in Python.

In the familiarization phase, participants saw a word or pair of words presented for 1 s just above the center of the computer screen. Immediately afterward, participants saw a single probe word presented just below the center of the screen and surrounded by question marks. The probe word could be one of the words just seen or another word from the same condition (pair or singleton). The participant had 1 s in which to press one of two keys to indicate whether the probe word was part of the chunk just shown or not. Immediate feedback was provided indicating whether their response was correct, incorrect, or too slow (>1 s). There were 176 familiarization trials during which each word was shown at least four times; twice at study and twice as a probe word. Probe words appeared at least once as a correct probe and once as a foil.

**Figure 1 fig1:**
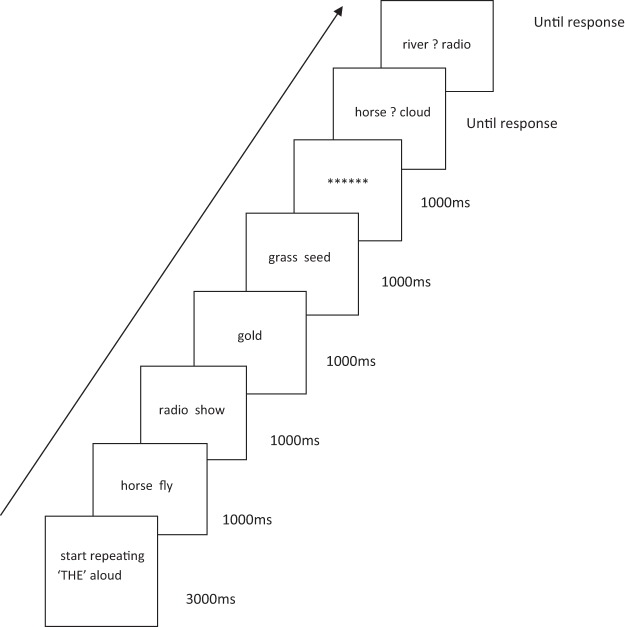
Presentation sequence for Experiments 1 and 2.

There was a brief articulatory suppression training session where participants were trained to repeat the word *the* aloud in time with a computer-presented metronome. This was followed by the list memory task (see Figure 1). Following the procedure of Cowan et al. (2012), all items were presented as chunks. That is, both words in the pair were displayed together, and pairs and singletons were both presented for 1 s. There were eight blocks of eight trials.[Fig-anchor fig1]

Immediately before presentation of the study list participants saw a message instructing them to begin performing articulatory suppression. They were required to say the word *the* aloud two times per second until the end of the lists which was indicated by a row of asterisks.

Memory for each word in the list was probed using a 2AFC recognition task. Each probe display consisted of a target word and a foil word separated by a question mark, and the task was to indicate which word had appeared in the list. Foil words were drawn from the same condition (single or pair) as the target word. The order of the probes was random subject to the constraint that items in pairs were not probed successively. There was a short break between blocks.

##### Results

In all the experiments reported here the primary question is whether the evidence favors the hypothesis that there is an effect of compression or the hypothesis that there is no effect of compression. This comparison of the relative merits of two alternative hypotheses is best achieved by computing Bayes factors (Kass & Raftery, 1995). Although all our conclusions will be based on Bayes factors, we also report standard frequentist analyses of variance (ANOVAs). In almost all cases where the Bayes factor favors the hypothesis that there is compression, the effect is also significant in the ANOVA. Conversely, when the Bayes factor favors the hypothesis that there is no compression, the corresponding effect in the ANOVA is nonsignificant. In describing the results, we use the verbal labels suggested by Jeffreys (1961) (e.g., a Bayes factor between 1/3 and 1/10 is substantial evidence for the *H*_0_ and a value between 1/10 and 1/30 is strong evidence). However, interpretation should be based on the value of the Bayes factor and not on the verbal label. Bayes factors were computed using the BayesFactor package in R.

**Figure 2 fig2:**
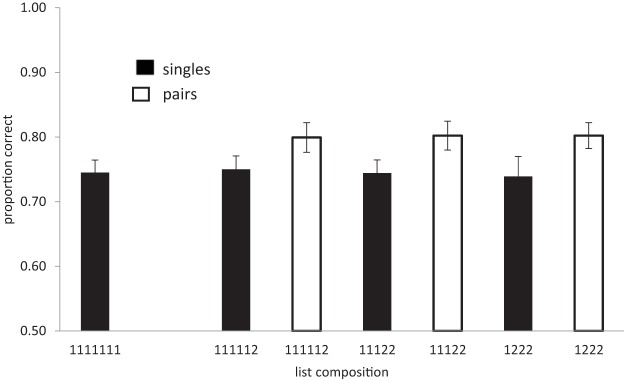
Experiment 1. Proportion of correct recognition responses to singles and pairs as a function of list composition. Error bars correspond to ±1 *SEM*. Proportion correct for lists with zero, one, two, or three pairs is .69, .71, .73, and .73, respectively.

The results of Experiment 1 are shown in Figure 2. We first performed an analysis of recognition accuracy of lists containing different numbers of chunks. There was decisive evidence for an effect of number of chunks (BF_10_ = 156; *F*(3, 81) = 7.58, *p* < .01), with lists comprised of fewer chunks (more pairs) being recalled better than lists with more chunks (fewer pairs and more singletons).[Fig-anchor fig2]

The critical question is whether having more pairs in a list improves the performance of the singles or pairs separately. For singles this analysis included all four list types, and for pairs only the three types containing pairs. In both cases the evidence favored the hypothesis that there was no compression (singles: BF_10_ = .05, strong evidence; *F* < 1; pairs: BF_10_ = .11, substantial evidence; *F* < 1): performance did not improve as the number of chunks in the list decreased.

Next, we examined the interaction between list type (number of chunks) and chunk size (singles-pairs). This was necessarily restricted to lists containing a mixture of both singletons and pairs, thus excluding lists containing seven singletons. There was decisive evidence for a main effect of chunk size (BF_10_ = 46,238; *F*(1, 27) = 14.3, *p* < .01), with a higher percentage of individual words in pairs being recalled than in singles. However, there was strong evidence that recall was unaffected by the number of pairs in the list (BF_10_ = .06; *F* < 1) and that list type (number of chunks) and chunk size (singles-pairs) did not interact (BF_10_ = .007; *F* < 1). Recognition of pairs remained around .8 across all list types and recognition of singletons remained around .73 across all list types.

#### Discussion of Experiment 1

A strong effect of chunking was evident in this experiment, with performance being better for lists containing more prelearned word pairs, and better for words from pairs than singletons. However, the benefit of chunking is entirely restricted to the pairs themselves. There is no effect of compression. The recall of singletons does not benefit from the presence of pairs elsewhere in the list. Furthermore, recall of the pairs themselves remains constant regardless of how many chunks are in the list. The recall benefit conferred by the presence of pairs is simply a direct function of the number of pairs in the list. This is exactly what we would expect if chunking was supported entirely by redintegration. Taken on its own, this result would lead us to exactly the opposite conclusion from Thalmann—there is no evidence that chunking is achieved by data compression.

Although Experiment 1 found clear evidence of chunking, perhaps the word pairs might not have been well enough learned to act as chunks in STM and support compression. Experiment 2 therefore includes an extra training phase to ensure that the chunks have been well learned. The extra training phase was based on the procedure used by Chen and Cowan (2005). The experiment is otherwise identical to Experiment 1. We incorporated extra training into all subsequent experiments other than Experiment 3, which was actually run before Experiment 2. Note that Experiments 1 and 3 were run using DMDX, which is unable to record the typed responses necessary for the extra training. To accommodate this requirement while keeping everything else identical, we ran the extra training phase using E-Prime (Schneider & Zuccolotto, 2002).

#### Experiment 2: 2AFC recognition with extra training

##### Participants

Twenty-eight members of the Cognition and Brain Sciences Unit Volunteer Panel completed this experiment (19 women, mean age 22.7 years). None had taken part in either of the first two experiments.

##### Materials and procedure

The materials and procedure for Experiment 2 were identical to that of Experiment 1 with the exception that there was an additional cued recall familiarization phase. On each trial, one word was presented in the center of the screen and the participant had to type the word with which it was paired or, type the letter *s* if it had been presented as a singleton. Feedback was given. This training phase continued with the repeated presentation of the entire set of stimuli until the participant was 100% correct on the set.

##### Results

**Figure 3 fig3:**
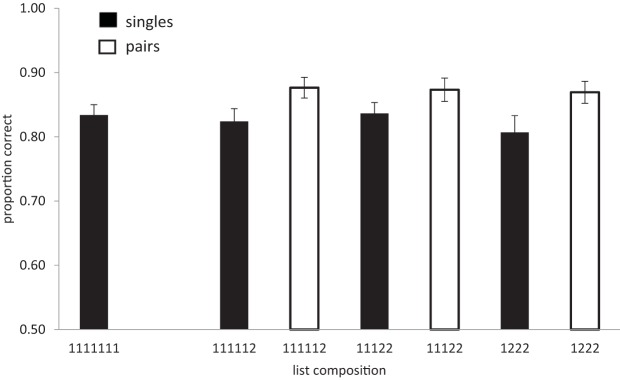
Experiment 2 (extra training). Proportion of correct recognition responses to singles and pairs as a function of list composition. Proportion correct for lists with zero, one, two, or three pairs is .83, .84, .86, and .86, respectively.

The results of Experiment 2 are shown in Figure 3. Although performance in Experiment 2 was better than performance in Experiment 1, the pattern of results remains the same: the data support the view that there is no compression. The error rate in the list memory task ranged between .03 and .41 (*M* = .142). An analysis of list type (number of chunks) showed that the beneficial effect of chunking was significant (BF_*10*_ = 4.9, substantial), *F*(3, 81) = 4.25, *p* < .01. accuracy: .83, .84, .86, .86, for 0, 1, 2, and 3 chunks. Singles and pairs were also analyzed separately. The Bayes factor for the analysis of number of chunks on singles alone was (BF_10_ = .25), *F*(3.81) = 1.5, and that for pairs alone (BF_10_ = .12), *F*(2, 54) = .18, which corresponds to substantial evidence for the null hypothesis. Next, we examined the interaction between list type (number of chunks) and chunk size (singles-pairs). This was necessarily restricted to lists containing a mixture of both singletons and pairs, thus excluding lists containing seven singletons. As in Experiment 1, performance was better for pairs than for singles, *F*(1, 27) = 30.5, *p* < .01; BF_10_ = 1 × 10^5^, but was not affected by the number of chunks (BF_10_ = .19; *F* < 1). There was no interaction between the number of chunks in a list and whether items were singles or pairs (BF_10_ = .04; *F* < 1).[Fig-anchor fig3]

#### Experiment 3: Serial recall

There are at least two reasons why we might not have found compression whereas Thalmann et al. (2019) did. First, we used a 2AFC procedure rather than serial recall. Second we used two-item chunks rather than three-item chunks. In the next experiment we examine chunking in an immediate serial recall task. The familiarization procedure was identical to that in Experiment 1. Here we used a standard serial recall paradigm where words were presented one at a time followed by spoken recall. In a pilot study we found that participants tended to say the pairs of items in chunks more rapidly than they would say two singletons. This fact alone might potentially alter the overall level of performance throughout the list by reducing the amount of time available for forgetting when the lists contained more chunks. We therefore adopted a paced recall procedure to force participants to recall the list at a rate of one item per second. Note that in Chen and Cowan’s (2005, 2009) serial recall experiments participants had to type their responses into a computer.

##### Participants

Twenty-eight members of the Cognition and Brain Sciences Unit Volunteer Panel completed the experiment (21 women, mean age = 21.6 years).

##### Materials and procedure

Stimuli were identical to those used in Experiment 1. In the serial recall phase participants received two practice trials followed by 64 test trials. In all list conditions, words were presented as single items rather than as chunks.

To initiate each trial the participant pressed the spacebar and repeated the word *the* aloud at the rate of two times per second. The first list item was presented 3 s later. List items were presented at a rate of one per second. The instruction “Prepare to recall” was then displayed on screen for 2 s and the participant had to stop speaking and prepare to recall the items aloud in the order they were presented. All responses were digitally recorded to be scored later. Participants were instructed to recall each item in synchrony with the appearance of seven visual cues (“X,” “XX,” “XXX,” and so on). The cues were presented for 750 ms with a pause of 250 ms in between. Participants were told to say “blank” for any item they could not recall in the appropriate position.

##### Results

The data were scored in three different ways. First they were scored according to the standard serial recall procedure where items are considered to be correct only when recalled in the correct serial position (strict scoring). A problem with this method is that a single omission at the start of the list leads to a score of zero even if all of the remaining items are recalled in the correct order. This is a particular problem for spoken recall. To overcome this limitation we also used a scoring procedure based on a Levenshtein edit distance metric (Kalm, Davis, & Norris, 2013; Kalm & Norris, 2016; Levenshtein, 1966), which counts the number of edit operations (insertions, deletions and substitutions) required to transform one sequence onto another. If recall is perfect, the Levenshtein edit distance will be zero. Kalm, Davis, and Norris used their Levenshtien Distance (LD) measure to score whole lists. However, we can extend the procedure to produce a score for each individual item. In calculating an edit distance there is frequently more than a single alignment between two sequences producing the same score. For example, if the list “1 2 3 4” is recalled as “1 2 4 3” the best alignment could be achieved by either deleting the 4 and inserting a 4 after the 3, or by deleting the 3 and inserting a 3 before the 4. The first two items are present on both of the best alignments and therefore score 1.0. 3 and 4 are each present on only half of the best alignments and are therefore both assigned a score of 0.5. This procedure provides a measure of the extent to which items are recalled in the correct order irrespective of position. Note that this implementation of the item-based procedure does not penalize individual items for the presence of insertions (extra items) recalled elsewhere in the list. We will refer to this as *Levenshtein scoring*. Mathy and Varré (2013) have used the more elaborate Needleman-Wunch string alignment method in their analysis of serial recall errors.

**Figure 4 fig4:**
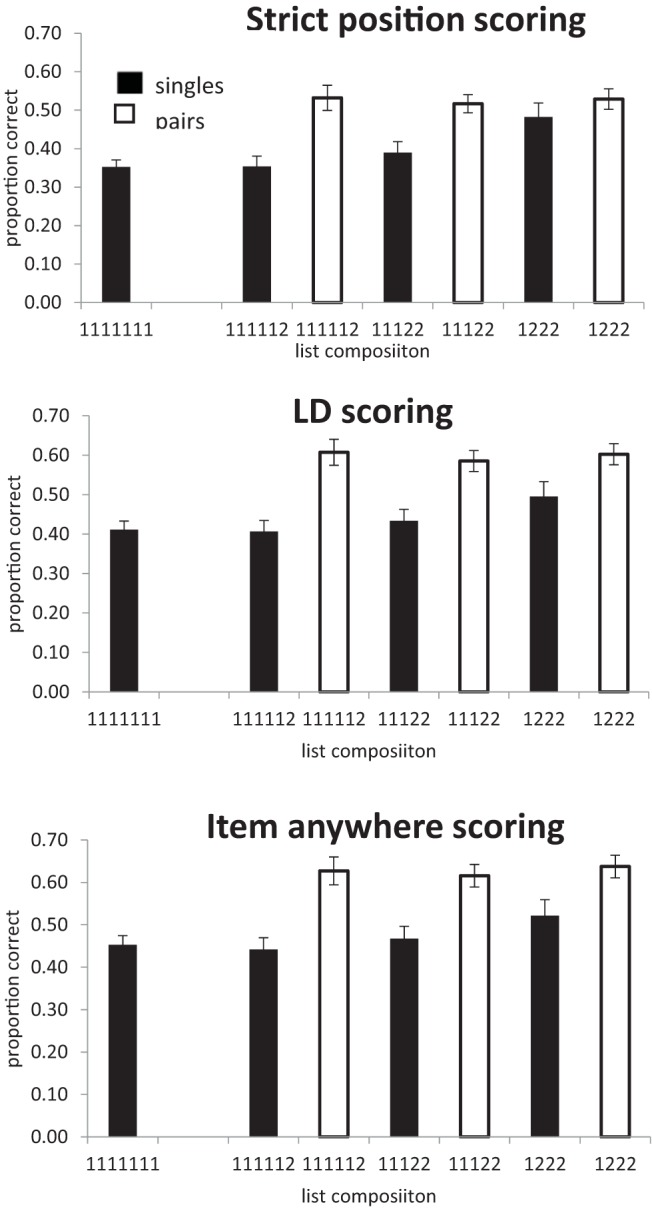
Experiment 3. Proportion of items correctly recalled as a function of list composition, scored as items in correct position, Levenshtein scoring, or items recalled regardless of whether they are in the correct position. Proportion correct for lists with zero, one, two, or three pairs is strict (.36, .41, .47, .53, respectively), LD (.42, .47, .52, .59, respectively) and item (.46, .50, .56, .63, respectively).

The third scoring procedure (item scoring) was the proportion of items correctly recalled regardless of position. (Chen & Cowan, 2005, termed this “lenient” scoring). The results of all three procedures are shown in Figure 4.[Fig-anchor fig4]

##### Items in correct position

All of the analyses reported here collapse the data over serial position. In the one-way analysis of list type (number of chunks) more items were recalled in the correct position as the number of pairs in the lists increased (BF_10_ = 9.5 × 10^14^), *F*(3, 81) = 49.0, *p* < .01. In the analysis of singles alone, recall also improved as the number of pairs increased (BF_10_ = 3.9 × 10^5^), *F*(3, 81) = 16.0, *p* < .0.1. For recall of pairs the evidence favored the null hypothesis that there was no effect of number of chunks on recall (BF_10_ = 0.13), *F*(2, 54) < 1. We also analyzed the three list types containing a mixture of both singles and pairs. Recall was better in list with fewer chunks (more pairs), (BF_10_ = 585), *F*(2, 54) = 9.9, *p* < .01, and was better for pairs than for singles (BF_10_ = 2.7 × 10^11^), *F*(1, 27) = 37.4, *p* < .01. There was also an interaction between list type (number of chunks) and chunk size (singles vs. pairs; BF_10_ = 31.6), *F*(2, 54) = 8.6, *p* < .01, which is attributable to the improvement in recall of singles when list contain more pairs. Performance on pairs differed by less than 0.02 across the three list types.

##### Levenshtein distance scoring

As with strict position scoring, the one-way analysis of list type (number of chunks) revealed that recall improved as the number of pairs in the lists increased (BF_10_ = 7.8 × 10^18^), *F*(3, 81) = 69.1, *p* < .01. However, in contrast to strict scoring, when considering only those lists containing a mixture of both single and pairs, there was little evidence that performance increased with when the number of pairs increased (BF_10_ = 1.25), *F*(2, 54) = 4.65, *p* < .05. Words in pairs were recalled better than singles (BF_10_ = 1.2 × 10^15^), *F*(1, 27) = 50.8, *p* < .01, but here was little evidence of an interaction between number of chunks and chunk size (singles vs. pairs; BF_10_ = 1.2), *F*(2, 54) = 3.5, *p* < .05.

Once again the Bayes factor for pairs alone indicated substantial evidence that memory for pairs was not influenced by the number of chunks (BF_10_ = .15), *F*(2, 54) < 1. However, for singletons the Bayes factor indicated that there was decisive evidence for an effect of number of chunks (BF_10_ = 56), *F*(3, 81) = 6.57 *p* < .01. Note that when analyzing just the singletons we can include data from all list types, including 1111111s, and so have more power than in the two-way analysis.

##### Items in any position

In the analysis of all four lists types, recall improved as the number of pairs increased (BF_10_ = 1.5 × 10^20^), *F*(3, 81) = 76.7, *p* < .01. In the analysis of only those lists containing a mixture of both singles and pairs there was little sign of an effect of number of pairs (BF_10_ = 1.3), *F*(2, 54) = 5.9, *p* < .01, although there was an effect of single/pair (BF_10_ = 1.0 × 10^16^), *F*(1, 27) = 57.8, *p* < .01. There was little evidence of an interaction between number of pairs and chunk size (singles vs. pairs. BF_10_ = .51), *F*(2, 54) = 2.1, *p* = .13. In the analysis of singles alone, there was decisive evidence for an effect of number of pairs (BF_10_ = 26.8), *F*(3, 81) = 58.7. In contrast, for pairs the evidence favored the hypothesis that there was no effect of varying the number of chunks (BF_10_ = .16; *F* < 1).

**Figure 5 fig5:**
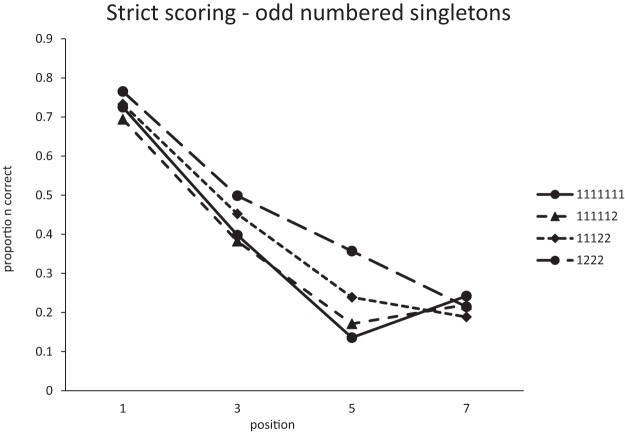
Experiment 3. Proportion of singletons correctly recalled in each position as a function of list composition.

An interesting insight into the improvement in recall of singles as the number of pairs increases can be gained from an examination of the serial position curves. Figure 5 plots the strict positional scores for singles. Positions 1, 3, 5, and 7 are the only positions where singles can appear in all list types. The improvement in recall as lists contain more pairs is almost entirely due to the improvement in recall at Position 5 in 1222 lists. Given that singles in 1222 lists can only appear in Positions 1, 3, 5, or 7, if participants either recall a pair correctly in Positions 6 and 7, or even simply know that there was a pair in Positions 6 and 7, the possible location of the single is significantly constrained. From Figure 4 we can see that item and LD scores are within a few percent of each other. If an item is recalled it is almost always recalled in the correct order relative to other items; item and order information seem to be closely coupled. Therefore, the most likely basis for the improvement in recall of singles with increasing number of pairs is that the presence of pairs provides more constraint on the recall of order information and that the order information is tightly bound to item information. Interestingly, this seems to be true of both singletons and pairs. One might have expected words in chunks to maintain their relative order and therefore be less sensitive than singletons to the difference between strict item and LD scoring.[Fig-anchor fig5]

A further hint that there is something different about recall of singles in 1222 lists can be seen by comparing the strict and LD scores for singles. Single scores are necessarily higher with LD scoring, but the difference between the two decreases as the number of pairs increases, implying that, with more pairs, singles are more likely to be recalled in the correct absolute serial position rather than simply in the correct relative order.

#### Discussion of Experiments 1–3

In all three experiments, lists containing more pairs were recalled better than singletons; there was a benefit of chunking. However, if that benefit were a consequence of data compression, a pair of items in a chunk should take up less memory capacity than a pair of singletons, and the benefit of chunking should extend to all items in the lists. Instead, in Experiments 1 and 2 the advantage of lists containing more pairs is entirely attributable to fact that the pairs themselves are remembered better then singletons. Recall of pairs and singletons is unaffected by the composition of the list.

This is exactly what is to be expected by a redintegration account, and contrary to what would be expected from a simple compression view such as Cowan et al.’s (2012) model VIII. If items in the chunk were replaced by their first word and that word was used as a pointer to the chunk stored in LTM, then each chunk in STM would take up as much capacity as a single word rather than two words, and this should free up capacity to remember one more word.

In Experiment 3, using a serial recall task, we see one part of the pattern we have taken to be the signature of compression; recall of singletons improves as the number of pairs in the list increases. However, interpretation of this effect is complicated by two factors. First, there is no improvement in recall of pairs as the number of pairs increases. If singletons show signs of compression, then pairs should too. The absence of a compression effect for pairs cannot be attributed to a ceiling effect as recall of pairs is only a little over .5 for strict scoring. Second, most of the improvement appears in lists where the presence of pairs constrains the location of singletons. Consequently, it is difficult to know whether this effect is a genuine effect of compression or a consequence of the constraints on position. If this result were simply due to improved positional information there should be no compression effect with item scoring. Items should be equally well recalled regardless of the number of chunks in the list but, with fewer chunks, items should be more likely to be recalled in the correct position. However, as already noted, the similarity between LD and item scoring tells us that when an item is recalled it is almost always recalled in the correct relative order.

A further important finding is that in mixed lists, pairs are always remembered better than singletons. In a simple chunking model, a pair and a singleton should behave identically; they are both chunks. Whether an item is remembered or forgotten depends on whether the chunk containing it is remembered or forgotten, and that should be the same regardless of the number of items in the chunk. It follows that recall of pairs and singletons should be equivalent, but they are not. Note that this was also the case in Thalmann et al.’s (2019) data. They also noted that this was not what would be expected by a simple compression model. One suggestion they made was that chunks might be better recalled because they are semantically more distinct than singletons. However, the superior recall of chunks follows necessarily from the redintegration view.

#### The cost of chunking

The results of Experiments 1–3 are not what would be expected on the basis of a simple compression model in which each chunk, regardless of size, occupies a single slot in memory. The fact that we see some indication of compression in Experiment 3 using serial recall might suggest that compression is specific to serial recall. This might possibly explain the discrepancy between our data from Experiments 1 and 2 and those of Thalmann et al. (2019).

However, another possible explanation for the apparent discrepancy between our data and that of Thalmann et al. (2019) is that chunking must come with a cost. Chunks in the input have to be recognized as such, recoded into a different form, and then be converted back into the same vocabulary as the input in order to be recalled. Bradmetz and Mathy (2008) and Portrat, Guida, Phénix, and Lemaire (2016) have both made proposals for how those costs might be simulated. One of the factors that determines whether the benefit of chunking outweighs the cost may be the size of the chunks; the capacity savings achieved by using a larger chunk may outweigh the cost of encoding and decoding those chunks. Indeed, several studies have demonstrated that chunking really does have a cost (Glanzer & Fleishman, 1967; Huang & Awh, 2018; Kleinberg & Kaufman, 1971; Pollack & Johnson, 1965).

Glanzer and Fleishman (1967) had participants recall sequences of nine binary digits presented simultaneously for 0.5 s. Prior to testing, three groups of participants were trained for 9 days to read nine-digit sequences. One group was trained to describe the binary digits as octal. A second group was trained to describe the digits in groups of three in English. For example, 110100000 would be read as “two ones, an oh; a one, two ohs; three ohs.” The final group was allowed to use a method of their own choice. After nine days of training one might have expected the binary to octal conversion process to become automatic. Based on the findings of Smith one might expect those trained to use the octal recoding strategy to perform best. However, they performed worst. Glanzer and Fleishman suggested that participants may not have been able to apply the strategy efficiently enough to benefit from it.

Pollack and Johnson (1965) also used a serial recall task but systematically varied the rate of presentation. They trained participants for twenty-eight 1.5-hr sessions to recode groups of four binary digits as the decimal numbers 0–15. They compared two presented at a constant rate and one where there were gaps between groups of four items. In both cases there was a large advantage of training at the 0.7 rate (ungrouped: 50%, grouped: 40%) which was greatly reduced at the 2.8 rate (12% and 9%). (These percentages were derived from their Figure 4 using WebPlotDigitizer https://automeris.io/WebPlotDigitizer/index.html).

Kleinberg and Kaufman (1971) tested memory for visual patterns. At fast presentation rates (less than one second) memory was constant in terms of amount of information, but memory became constant in chunks at slower rates. That is, effective use of chunking required time.

Huang and Awh (2018) examined the time to decode chunks. They used a probe paradigm similar to that of Brady et al. (2009). Stimuli could either be four color pairs or four letter pairs, and pairs were either familiar or not. In the case of letters the familiar pairs formed words. Performance was better with familiar pairs, but this advantage disappeared when participants had to respond under time pressure.

These studies highlight the fact that the benefit of chunking has to be weighed against the cost of recoding and decoding. In the next three experiments we therefore tried to increase the potential benefit of recoding items into chunks by familiarizing participants with larger chunks—three words rather than two.

### Experiments 4–6: Three-Item Chunks

#### Experiment 4: 2AFC with triples

In this experiment we asked whether moving from two-item chunks to three-item chunks might also generate evidence for compression in a probe recognition task.

##### Materials

Stimuli were based on 45 triples and list could have one of the following structures: 1111111, 11113, or 133. The composition of the lists is shown in Table 3. Stimuli were based on the 45 triples shown in the Appendix. For each participant nine of those triples were selected at random to act as triples and one randomly selected item from each of the remaining 36 were used as singletons. In the familiarization trials each of the nine triples appeared six times; three times so that each word could be probed, and three times as a foil where the probe was a different word. Each singleton also appeared six times, once with an identical probe, and once with a foil. This experiment incorporated the same cued recall training phase as in Experiment 2 with the exception that for the triples, participants had to type in the remaining two words of the set. During the presentation phase items were presented triples were presented simultaneously. There were eight blocks of nine trials. As before, participants had to perform articulatory suppression during list presentation and recall was paced.[Table-anchor tbl3]

##### Participants

Thirty members of the Cognition and Brain Sciences Unit Volunteer Panel participated in the experiment. The data from one participant was lost because of a technical problem and another was replaced as they took 18 iterations through the training phase to reach criterion. The analyses are therefore based on 28 participants (22 women, *M*_age_ = 22 years).

##### Results

**Figure 6 fig6:**
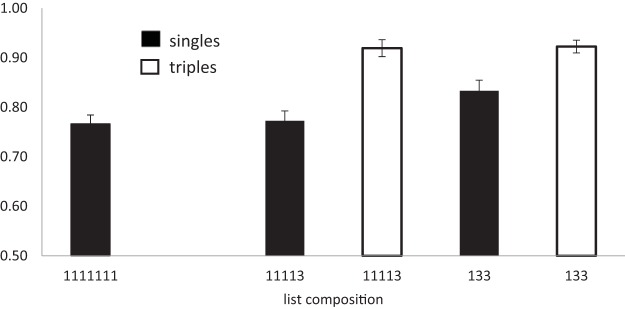
Experiment 4. Proportion of correct recognition responses to singles and triples as a function of list composition.

The results are shown in Figure 6. As with all previous experiments performance increases as the number of multiword chunks increased, *F*(2, 54) = 69.6, *p* < .001, BF_10_ = 3.9 × 10^12^. In contrast to probe recall with pairs (Experiments 1 and 2), an analysis of singles alone showed that performance improved as a function of the number of triples in the list (BF_10_ = 39.5), *F*(2, 54) = 8.2 *p* < .001. However, any benefit of adding an extra triple is seen only with 133 lists; scores for 11113 lists were almost identical to scores for 1111111 lists. In the analysis of triples alone, there was no indication of any improvement in recall as a function of number of triples in the list (BF_10_ = 0.28; *F* < 1). This could be a ceiling effect.[Fig-anchor fig6]

In the analysis of the lists containing both singletons and triples (11113 and 133) there were effects of chunk size (singles vs. triples; BF_10_ = 7.6 × 10^11^), *F*(1, 27) = 88.6, *p* < .001, and the number of triples in the list (BF_10_ = 6.9), *F*(1, 27) = 7.14, *p* < .05. In line with the separate analyses of singles and triples there was also an interaction between the two (BF_10_ = 2.3), *F*(1, 27) = 5.1, *p* < .05, such that singles benefitted more from the presence of more triples than did the triples themselves.

##### Discussion

The use of three-item chunks changed the pattern of findings in one important respect: performance on singletons improved as the number of triples in the list increased. This is exactly what would be expected if chunking led to data compression. However, there was no variation in recall of triples as a function of the number of triples in the list. Although a data-compression view would predict that triples should also benefit, any advantage of having two triples in a list rather than one may have been obscured by a ceiling effect as performance on triples was above 0.9. As in the previous experiments, there was an overall benefit of chunking, with words in triples being recalled much better than singletons. This is not what would be expected from a simple slot model. In the next experiment, we examine whether the move from pairs to triples also reveals evidence of compression in serial recall.

#### Experiment 5: Serial recall with triples

The procedure for Experiment 5 was identical to that for Experiment 3 apart from the fact that, to improve performance, the lists contained only six items and we used the more extensive training procedure used in Experiments 2 and 4. Lists could consist of six singletons, three singletons and a triple, or two triples. The number of words presented here was always six, but the number of chunks varied as in Table 4:[Table-anchor tbl4]

##### Participants

Thirty members of the Cognition and Brain Sciences Unit Volunteer Panel completed this experiment. None had taken part in any of the first three experiments. Two participants who took eight iterations through the extra training to reach criterion were excluded. The analyses are based on the remaining 28 participants (16 women, *M*_age_ 20.5 years).

##### Materials and procedure

This experiment was similar to Experiment 3 with the addition of a cued recall phase as used in Experiments 2 and 4. As lists were only six items long, the stimuli were constructed around a set of 36 word triples (see the Appendix). As in Experiment 4, for each participant nine triples were selected at random to be used as complete triples and one word from each of the remaining 27 triples was used as the singleton.

##### Results

**Figure 7 fig7:**
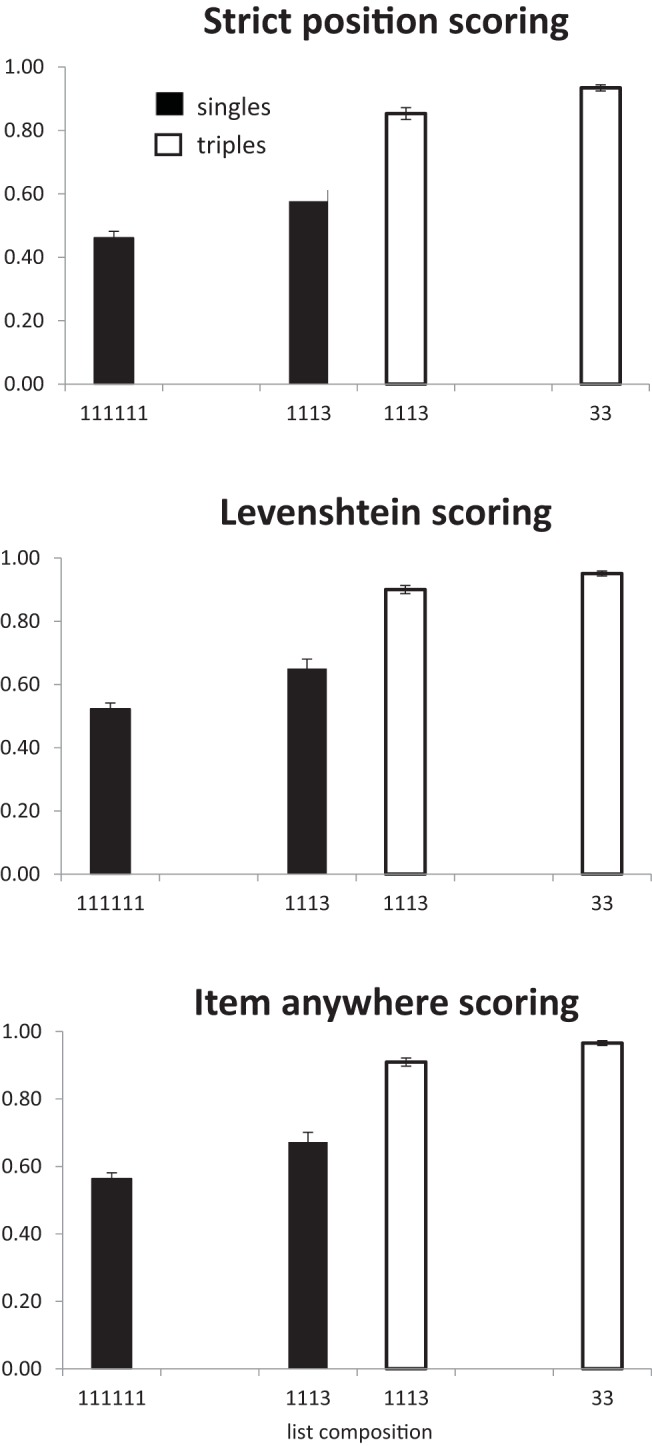
Experiment 5. Proportion of items correctly recalled as a function of list composition, scored as either items in correct position, Levenshtein scoring, or items recalled regardless of whether they are in the correct position. Proportion correct for lists with zero, one, or two triples is .77, .84, and .91, respectively.

**Figure 8 fig8:**
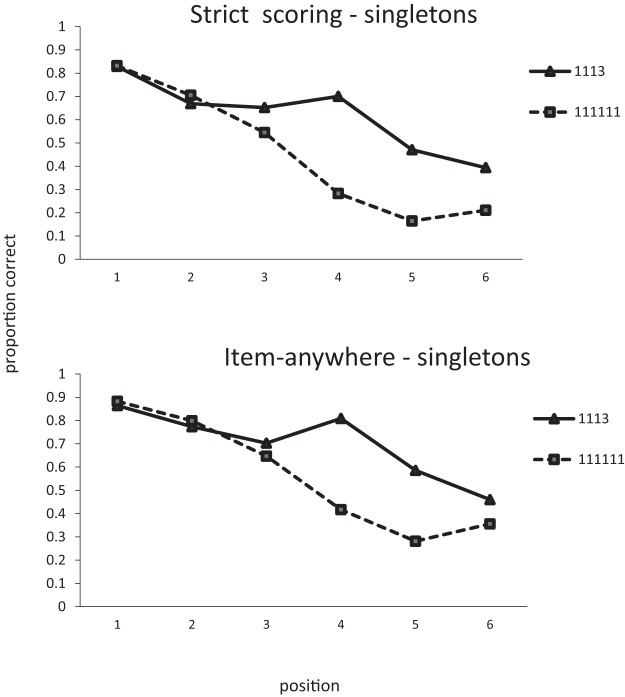
Experiment 5. Proportion of singletons recalled at each position as a function of serial position.

The analysis follows the same procedure as Experiment 3. The data are shown in Figures 7 and 8.[Fig-anchor fig7][Fig-anchor fig8]

###### Items in correct position

There was an overall effect of chunking with the number of items being recalled in the correct position increasing as the number of triples increased (BF_10_ = 6 × 10^33^), *F*(2, 54) = 410, *p* < .01. In a separate analysis of the 1113 condition there was a main effect of chunk size with more triples being recalled in the correct position than singletons (BF_10_ = 1.3 × 10^9^), *F*(1, 27) = 112, *p* < .01. In an analysis of the singles in the 111111 and 1113 conditions there was a significant effect of list type, with more single items being recalled in the correct position in the four chunk lists (1113) than the six chunk (111111) lists (BF_10_ = 1.8 × 10^5^), *F*(1, 27) = 55.2, *p* < .01. Similarly, triples in 33 lists were recalled better than triples in 1113 lists (BF_10_ = 3850), *F*(1, 27) = 31.5, *p* < .01. Note that in this experiment it is not possible to perform a two-way analysis of item-type and number of chunks because 1113 is the only list-type that contains both singles and triples.

###### LD scoring

As with strict scoring, there was main effect of list type (BF_10_ = 4.1 × 10^35^), *F*(2, 54) = 477, *p* < .01. In the 1113 condition there was an effect of chunk size (BF_10_ = 4.1 × 10^9^), *F*(1, 27) = 98.7, *p* < .01. Singles benefited from the presence of a triple—11111 vs. 1113: BF_10_ = 7.4 × 10^4^; *F*(1, 27) = 50.3, *p* < .01—and triples were better in 33 lists than in 1113 lists (BF_10_ = 397), *F*(1, 27) = 21.8, *p* < .01.

###### Items in any position

There was an effect of list type, with more items being recalled as number of chunks decreased (BF_10_ = 2.76 × 10^34^), *F*(2, 54) = 410.6, *p* < .01. In the 1113 condition more words were recalled from triples than singles (BF_10_ = 1.6 × 10^10^), *F*(1, 27) = 89.2, *p* < .01. Singles in 1113 lists were recalled better than singles in 111111 lists (BF_10_ = 6384), *F*(1, 27) = 36.6, *p* < .01. Triples in 33 lists were recalled better than triples in 1113 lists (BF_10_ = 3617), *F*(1, 27) = 29.8, *p* < .01.

##### Discussion

The most notable feature of the present experiment compared to Experiment 3 (serial recall of pairs) is that the overall level of performance is substantially higher. The strict position score of the pairs in the 1222 chunks was 0.53 compared with 0.93 for the 33 triples here. The combination of the larger chunks and the shorter lists both acted to boost performance. Note that the improved performance is unlikely to be solely attributable to the use of a shorter list. The improvement on lists of pure singles between the experiments was about 0.1, whereas the difference between the 2221 and 33 condition was 0.4. That is, three-quarters of the improvement seems to have come about through the use of bigger chunks.

As in Experiment 4, singletons are recalled better when there is a larger chunk in the list. Triples are also recalled slightly better when the rest of the list forms a single chunk rather than three chunks (singletons). It is impossible to make any quantitative comparisons between the effects of the number of chunks on singles and triples because performance on triples is near ceiling.

The beneficial effect of having other chunks in the list holds regardless of the scoring we use. This is exactly what is expected if participants are able to form a compressed memory representation of the chunks. If chunks take up less memory capacity than singletons, then that capacity should be available to store more information about singletons. Chen and Cowan (2005, 2009) only found evidence for constant chunk capacity using a lenient serial order scoring procedure. Given that our lists are all the same length, our data cannot speak to the issue of whether there is a fixed chunk-based limit, but it is interesting that we see signs of compression even when the data are analyzed using strict scoring.

So far we have had our participants perform articulatory suppression during list presentation. We did this because these are the conditions under which Chen and Cowan (2009) had found the strongest evidence for chunk-based memory. However, most studies of chunking have not used suppression. Thalmann et al. (2019) used suppression only in their Experiment 3. Chen and Cowan (2009) motivated their use of suppression by suggesting that by preventing phonological rehearsal “What remains is a core verbal working-memory capacity” (p. 1420). However, at least within the Baddeley and Hitch (1974) working memory framework, articulatory suppression also prevents visual material being recoded into the phonological store. Chen and Cowan’s definition of core verbal working memory would therefore exclude the phonological store, which is probably the most widely studied component of verbal STM. Core verbal-working memory would seem to correspond to that part of verbal working memory outside of the phonological store. In the absence of articulatory suppression, immediate serial recall will be largely mediated by the phonological store rather than ‘core verbal working memory’ or the episodic buffer. In everyday life we rarely find ourselves trying to remember while performing suppression. The next experiment therefore dispenses with articulatory suppression in an attempt to discover whether chunking might also enable compression when the phonological loop is involved.

#### Experiment 6: Serial Recall with Triples Without Articulatory Suppression

##### Procedure

This experiment differed in two respects from Experiment 5. First, participants were not required to perform articulatory suppression. The instruction to begin suppressing was replaced with “Ready.” Second, because performance is always better without suppression than with, and we had already run into ceiling effects with triples in Experiment 5, we reverted to using seven-item lists structured as in Experiment 4. Although the intention was to ensure that both singletons and triples appeared equally often at all possible locations, a programming error meant that no triples began at position 5 that is, no lists ended in a triple.

Twenty-eight members of the Cognition and Brain Sciences Unit Volunteer Panel completed the experiment (21 women, *M*_age_ = 21.5 years). None had taken part in any of the previous experiments.

##### Results

**Figure 9 fig9:**
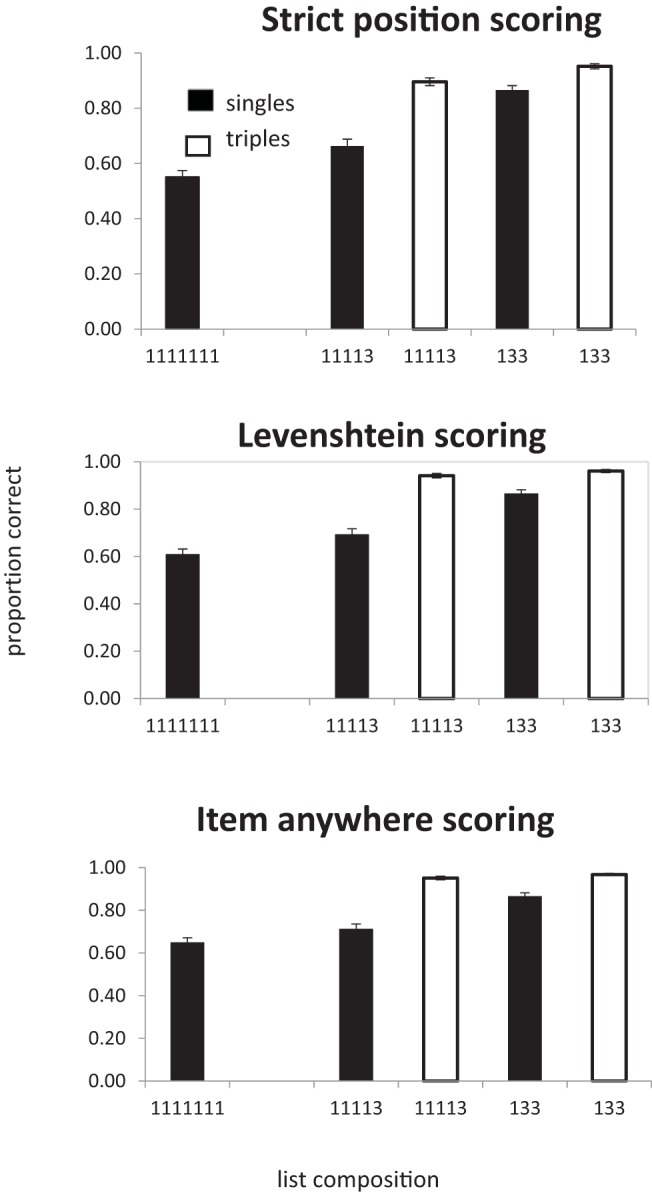
Experiment 6. Proportion of items correctly recalled as a function of list composition, scored as either items in correct position, Levenshtein scoring, or items recalled regardless of whether they are in the correct position. Proportion correct for lists with zero, one, or two triples is strict (.55, .76, and .94, respectively), LD (.61, .80, and .95, respectively), and item (.65, .82, and .95, respectively).

The results are shown in Figure 9. The main findings are consistent across the different scoring procedures. There was a main effect of number of chunks in a list, with performance being better for lists containing more triples—strict: *F*(2, 54) = 277.08, *p* < .01, BF_10_ = 5.1 × 10^28^; LD: BF_10_ = 5.9 × 10^28^; *F*(2, 54) = 276.96, *p* < .01; and item: BF_10_ = 1.4x 10^26^; *F*(2, 54) = 227.3, *p* < .01. In the analysis of singles only, performance increased as the number of triples increased—strict: BF_10_ = 1.8x 10^20^; *F*(2, 54) = 147.6, *p* < .01; LD: BF_10_ = 3.2 × 10^17^; *F*(2, 54) = 114.8, *p* < .01; and item: BF_10_ = 3.7 × 10^14^; *F*(2, 54) = 84.9, *p* < .01. In the analysis of triples alone (11113, 133), only strict scoring produced substantial evidence that increasing the number of triples improved performance—strict: BF_10_ = 132; *F*(1, 27) = 16.28, *p* < .01; LD: BF_10_ = 1.46; *F*(1, 27) = 3.78, *p* = .06; and item: BF_10_ = 1.3; *F*(1, 27) = 3.63, *p* = .07.[Fig-anchor fig9]

Only two list types contain both singles and triples (11113, 133). In an analysis of these lists triples were better recalled than singletons—strict: BF_10_ = 2.0 × 10^20^; *F*(1, 27) = 118.8, *p* < .01; LD: BF_10_ = 2.4 × 10^23^; *F*(1, 27) = 136.83, *p* < .01; and item: BF_10_ = 7.0 × 10^23^; *F*(1, 27) = 137.9, *p* < .01—and recall was better in the list with more triples (133 compared to 11113—strict: BF_10_ = 1.4 × 10^16^; *F*(1, 27) = 130.7, *p* < .01; LD: BF_10_ = 4.2 ×10^12^; *F*(1, 27) = 128, *p* < .01; and item: BF_10_ = 1.1 × 10^11^; *F*(1, 27) = 71.1, *p* < .01. There was an interaction between chunk size and list type, with the effect of number of chunks being greater for singletons than triples—strict: BF_10_ = 2 × 10^20^; *F*(1, 27) = 51.1, *p* < .01; LD: BF_10_ = 8.3 × 10^5^; *F*(1, 27) = 61.2, *p* < .01; and item: BF_10_ = 1.7 × 10^5^; *F*(1, 27) = 53.9, *p* < .01.

##### Discussion

As in Experiment 5, the data here show the pattern we have taken to be a signature of compression - performance on singles improves when there are more triples in the list. This effect does not, therefore, seem to depend on whether or not participants perform articulatory suppression. However, as in all of the previous experiments, items in larger chunks (triples) are recalled better than items in smaller chunks (singletons). This is not what would be expected from a simple compression model. This result differs from Experiment 2 of Chen and Cowan (2005) which used eight-item lists without articulatory suppression. With strict scoring they found no difference between lists formed from eight singletons or eight pairs. However, they did find a difference with item (lenient) scoring.

#### Serial position data from Experiments 5 and 6

The results of Experiments 4, 5, and 6 seem to provide clear evidence for some form of compression; memory for both singletons and triples improves when there are more triples in the lists. This is consistent with the chunking model preferred by Cowan et al. (2012). However, according to that model there should be no difference in the probability of recalling a singleton or a triple. In effect, this chunk-based model is an instantiation of Conrad’s (1965) slot model. There is a set of discrete slots and each slot can hold a single chunk. However, although a simple slot model might be applicable to visual STM experiments where all items are presented simultaneously followed by a single probe (e.g., Brady et al., 2009; Luck & Vogel, 1997), they become implausible as models of verbal STM where words must be processed one at a time. In a standard serial recall experiment items must necessarily be both presented and recalled in sequence.

**Figure 10 fig10:**
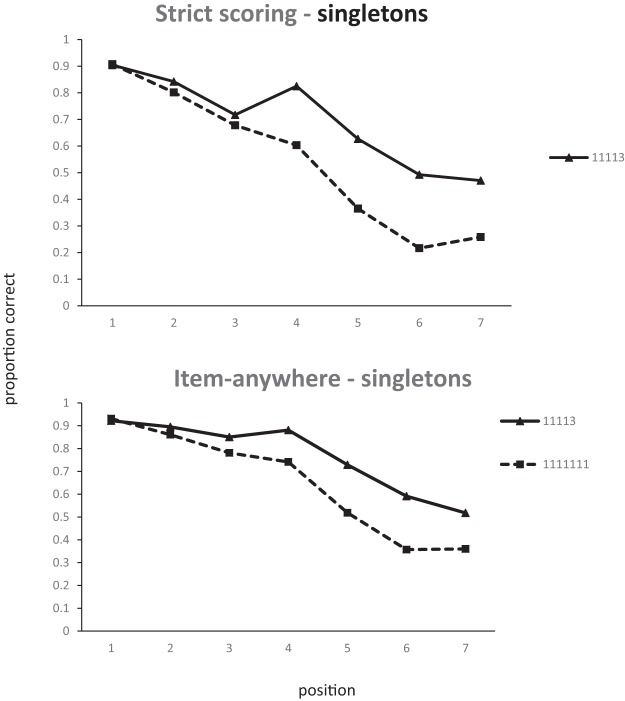
Experiment 6. Proportion of singletons recalled at each position as a function of serial position.

In Cowan et al.’s (2012) model, all of the chunks presented need to be held in memory in order to select the *N* chunks that will finally be stored. Alternatively, the first *N* chunks would be encoded and any further items would not enter into memory. This would seem to predict that only the first *N* items would ever be recalled. Such a model would have problems explaining why there should ever be a recency effect. Items toward the end of a supraspan list should simply never be stored in STM. If simple slot models are unable to account for the basic data on serial recall, how can we explain the advantage of chunking seen in Experiments 4–5? We can get some insight into what is happening by looking at the serial position curves. Figure 10 shows composite serial position data for singletons in lists containing either only singletons or singletons and just one triple. In the [1]1113 conditions, the first three points correspond to items occurring before the triple. Item one averages over lists where triples appear in Positions 2, 3, and 4. Item two averages over lists where triples begin at Positions 3 and 4, and, for Item 3, the triple always starts at Position 4. The difference between the two conditions is largely restricted to the latter half of the list. That is, the benefit of having a triple in the list applies mainly to those items occurring after the triple. Thalmann et al. found exactly the same effect in their data—the main benefit if chunking was found when chunks appeared at the start of the list. A simple compression account should predict that all list items would benefit from compression. It should make no difference what order the items appear in.[Fig-anchor fig10]

Consider what might happen when processing a 1113 list where the singletons appear in the first three position. Until the fourth item appears, this list is effectively identical to the initial items in a list of six singletons. The initial encoding of these items should therefore be unaffected by the presence of a later chunk, even though that later chunk would be expected to take less memory capacity than when the remaining items are all singletons. A simple compression view might suggest that this extra capacity could be used to enhance memory (or reduce forgetting) of the earlier items. However, this would imply that the list items already in memory could be somehow be revisited and then reencoded in memory.

Given these problems with slot models of verbal memory it should not come as a surprise that there are no current computational models of verbal STM that use a fixed set of discrete slots. These models have softer constraints on capacity which are imposed by factors such as decay (Page & Norris, 1998) or interference (Farrell & Lewandowsky, 2002). Supraspan items are always encoded to some extent. Each additional item impairs performance of all of the items in the list either by interfering with the representations of those items, or by adding extra time for time-based decay. In these models the simplest chunk-based account would be that there is no change in the encoding of single items as a function of the presence of chunks at all; chunks just behave like single items. This will confer a benefit to single items following chunks but not before. If a chunk is encoded as a single item, then an item occurring immediately after a three-item chunk should effectively be recalled as well as the item in Position 2 of a list of singletons. Both items are the second chunk in the list. This is roughly what we see in both the item and order data where Items 3, 4, and 5 in the 1113 lists are recalled at the same level as Items 2, 3, and 4 in the 111111 list. The most consistent result from all six experiments is that multiword chunks are remembered better than singletons. As noted earlier, this is not what would be expected on the basis of a simple chunking model that took no account of serial recall. In such a model recall of singletons and multiword chunks should be equivalent. However, this is not what is expected from models of serial recall. The first item in a chunk should be recalled with equivalent accuracy to a singleton in the same position. However, recall of subsequent singletons will decrease as a consequence of decay or interference, but all items in a chunk will be recalled together with the same accuracy as the first item. Memory for chunks will therefore be superior to memory for singletons. We now report a series of simulations to determine whether we can provide a quantitative account for our results by incorporating this simple assumption into an existing model of serial recall. The aim of these simulations is to establish whether this verbal explanation can give a quantitative account of the data.

## Simulations

Here we use the primacy model of Page and Norris (1998) as an example of a simple model able to give an account of the main benchmark features of serial recall. In common with most models of serial recall its behavior relies on the combination of a primacy gradient, response suppression, and a mechanism whereby information is lost from memory (see Lewandowsky & Farrell, 2008, for an analysis of the features of different models of STM). Given that the assumption we are making about chunking is so simple, other models should also have little difficulty simulating the same data. Our aim in using the primacy model is simply to demonstrate that the pattern of data we see when chunks and singletons are intermixed can be readily accommodated by the principles shared by most computational models of serial recall from verbal STM.

In the primacy model, information is assumed to be lost due to time-based decay. However, for the present purposes it makes no difference whether information is lost because of decay or because of interference. One appealing feature of the primacy model is that it captures the simple idea that the discriminability of order information and the accessibility of item information decrease during recall of the list. It starts by setting up an activation gradient whereby the first item has given level of activation and successive items have one unit less activation. Recall is performed by adding zero-mean Gaussian noise to the activations and choosing the item with the largest activation. For the first item in the list the most likely item to be recalled will therefore be the first item, with the second item being less likely to be recalled, and subsequent items with even lower activation being even less likely to be recalled. The most active item then has its activation suppressed and will not be recalled again. The chosen item then has further noise (omission noise) added to its activation and the resulting value is compared with an omission threshold. If the activation exceeds the threshold the item is recalled, if not, it is omitted. That chosen item then has its activation suppressed and will not be recalled again. After each recall cycle the activation gradient is made to decay by multiplying it by a decay factor. Consequently, the activation levels of the remaining items are reduced and move closer together and therefore become more subject to being recalled out of position, or being omitted.

The model therefore has five parameters: peak activation, selection noise, omission noise, omission threshold, and decay. The simulations^3^ incorporate the assumption that each triple is treated single item. As is the case with Cowan et al.’s (2012) mathematical models, we are not making any claims about the exact representation of a chunk in STM. One straightforward consequence of this assumption is that all three items in any triple are always either recalled or omitted together and hence give rise to the same recall score. This is a very close approximation to what we see in the data if we examine lists with a single triple. In Experiment 5, if any one member of a triple was recalled, the probability that all three members would be recalled was .94. In Experiment 6, this probability was .97. Recall of triples as a function of the starting point of the triple are shown in Tables 5 and 6. The fact that all members of a triple are always recalled together means that the model necessarily predicts that chunks will be better recalled than singletons. This is the most consistent feature of the data in all six experiments. In a three-item chunk all words in the chunk will be recalled with the same accuracy. With three singletons in the same positions, the first item will be recalled at the same level as the first item in the chunk but recall of the following two items will decline. Overall, triples will therefore be recalled better than singletons.[Table-anchor tbl5][Table-anchor tbl6]

**Figure 11 fig11:**
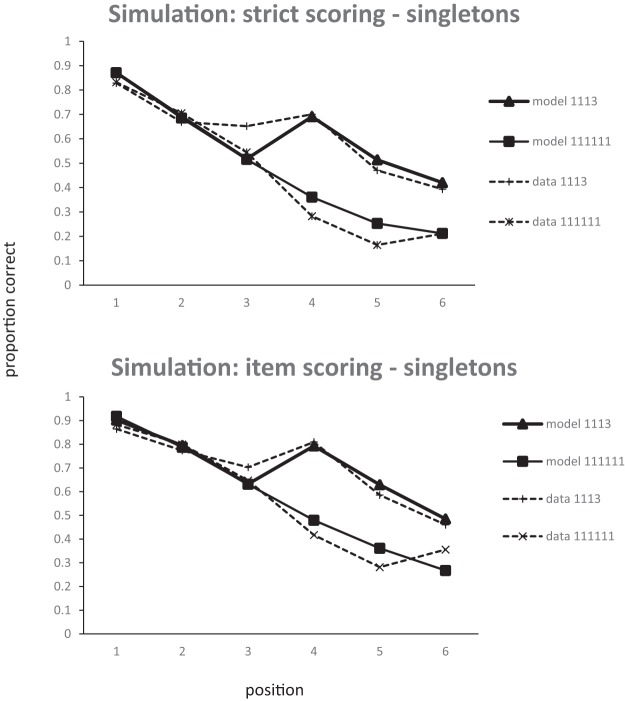
Simulations of recall of singletons in Experiment 5 using the primacy model.

**Figure 12 fig12:**
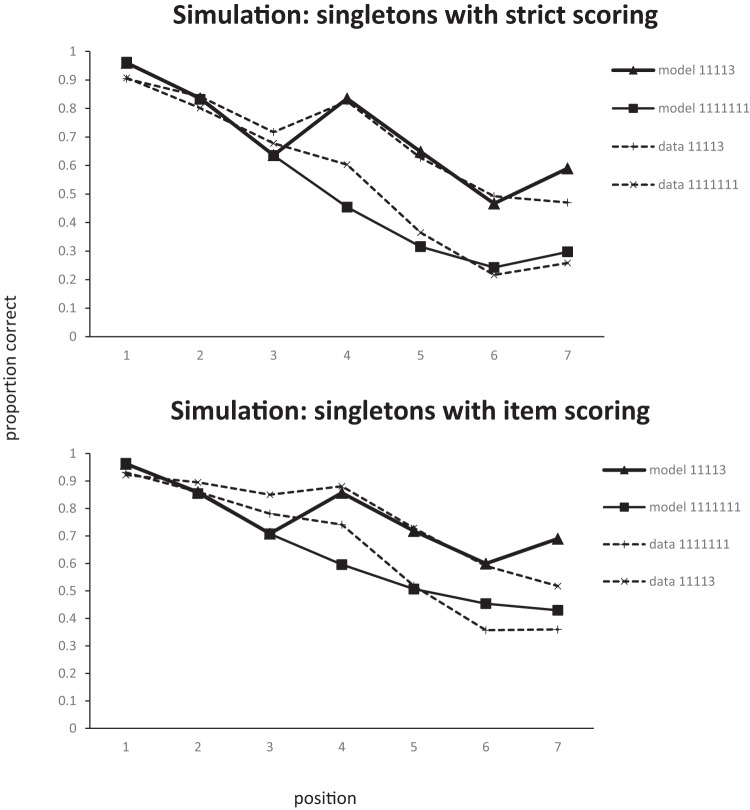
Simulations of recall of singletons in Experiment 6 using the primacy model.

**Figure 13 fig13:**
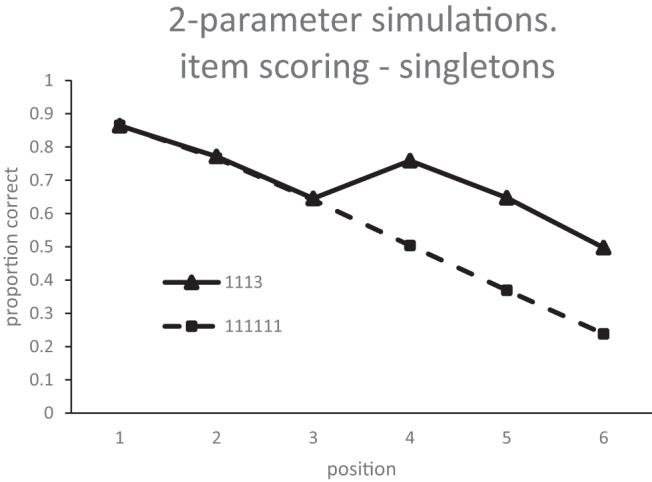
Simulations of recall of singletons in Experiment 5 using a two-parameter version of the primacy model.

The serial position curves produced by fitting the model to item scores and strict position scores simultaneously (i.e., 24 data points in Experiment 5, 28 in Experiment 6) are shown in Figures 11 and 12. Fitting was performed using Powell’s (1964) conjugate gradient method and each step of the optimization process was based on 50,000 iterations through the model. With this many iterations, the Root Mean Squared (RMS) error of the model varies by no more than 0.001 between steps with the same parameters. The model does an excellent job of capturing the data for both Experiment 5 (RMS error: .053) and Experiment 6 (RMS error: .07). In fact, given that the pattern of data is mainly carried by omissions, most of the data can be captured by an even simpler two-parameter model. The simulation of the data from Experiment 5 in Figure 13 has the peak step and omission noise as free parameters, fixes the omission threshold at zero, and has no decay. Of course, the full primacy model simulation is to be preferred as it has the advantage that it simultaneously simulates order errors as well as item errors.[Fig-anchor fig11][Fig-anchor fig12][Fig-anchor fig13]

One interesting feature of these simulations is that by assuming that each chunk is treated as a single item we are implicitly assuming that forgetting during recall is the same for a chunk as it is for a single item. The simulations make no allowance for the fact that presentation and recall of a chunk will take longer than a single item and might produce more decay or interference for subsequent items. However, decay and interference models would seem to predict that forgetting should be a function of either time or items, and not of chunks.

We examined this by fitting a variant of the model where there were three cycles of decay per triple. Although the triple was treated as a single item, we assumed that each member of the triple would have the same effect on decay of the remaining items as a singleton. This produced only marginally poorer fits but did so by setting the decay to be close to 1.0. This means that the decrease in performance over position was carried entirely by the increase in omissions. The increase in omissions over position is produced by the activation gradient at encoding, which decreases by one step per item or chunk. As the activation decreases, so the opportunity for omissions increases (the activation plus the omission noise will fall below the omission threshold). We can also investigate whether the fit of the model will change if we allow the activation step for a chunk to be larger than for a singleton. If we add a parameter to the model that allows the reduction in activation caused by a chunk to vary, we find that in Experiment 5 the activation step for a chunk can be up to 1.5 times that for a singleton with no change in the goodness of fit. These additional simulations show that the good model fits we obtain when triples and singletons are treated identically should not be taken as definitive evidence that triples must be identical to singletons. There is room to vary the model parameters without any change in the degree of fit.

A further point to note is that the simulations embody the assumption that all chunks are coded as such. That is, unlike the model favored by Cowan et al. (2012) for their data, none of the chunks degenerate and become represented as two or three singletons instead. Combining these two features of the model together it should be apparent that it has to predict that memory capacity for three triples would be the same as for three singletons. This contrasts with Chen and Cowan’s (2005, 2009) report that a constant chunk-based capacity was found only with articulatory suppression. Here we see a similar pattern of data regardless of whether or not there is suppression. Furthermore, our simulations, which are based on the assumption that a chunk is indeed equivalent to a singleton, fit the data from Experiments 5 and 6 equally well.

It is important to note that although the model treats chunks as the storage units of memory it does not have a hard chunk-based limit on capacity. As noted earlier, the softer constraints in this and other models of verbal STM are necessary to capture fundamental properties of serial recall such as the shape of the serial position curve. Of course, the simulations we have presented here are not intended as a complete model of chunking in verbal STM. Instead they indicate the simplest possible set of assumptions necessary to account for our serial recall data. Although the simulations of serial recall suggest that chunks are treated in the same way as singletons, there is no comparable model of the 2AFC task that would allow us to estimate whether chunks are treated like singletons in that task too. Sometimes capacity in 2AFC is measured using Cowan’s k (Cowan, 2001). However, this is based on the assumption that there is a fixed-capacity chunk-based storage. Cowan et al. (2012) found that capacity was not constant in the way that this simple model would assume. Instead of remaining constant, k decreased as chunks contained more items.^4^ However, this need not imply that what is stored in memory is different in serial recall and 2AFC. This decrease is what one might expect if, for example, there is any cost at all in decomposing a chunk to determine whether it contains the probe item.

Thalmann et al. (2019) took the finding that chunks only had a benefit when they appeared early in the list as evidence against decay. Given that we successfully simulated the data with a model that is neutral as to whether loss of information form STM is by decay or interference, it is hard to see how this finding itself could possibly be inconsistent with decay models. They suggest that a chunked representation should take less time to rehearse than three unchunked items and that this should benefit items before a chunk as well as afterward. However, this explanation rests on two questionable assumptions. The first is that what is rehearsed is a representation of the chunk and not its constituent items. This would only be possible if the phonological representation of the chunk was replaced by say, the first word in the chunk and that participants rehearsed that word rather than the whole chunk. However, the second assumption is that what is rehearsed is the entire list and that it continues to be rehearsed after the final chunk is presented. However, in their primacy model which incorporates both decay and rehearsal, Page and Norris (1998) assumed that rehearsal is performed cumulatively from the beginning of the list but is abandoned at the point where all items can no longer be rehearsed in the interval between items. Given that items in our experiments and in Thalmann et al. (2019) were presented at a rate of one per second, participants are unlikely to be able to rehearse more than four items in the gap between items. They will therefore have abandoned rehearsal well before the end of the list.

## General Discussion

Chunking is usually thought of as a way of squeezing more information into a limited capacity STM system. That is, chunking is a form of data compression. Currently there are only two studies (Brady et al., 2009; Thalmann et al., 2019) that provide support for the idea that representations in STM may become compressed so as to allow more information to be stored in memory. The data from Thalmann et al. (2019) et al. provide convincing evidence that chunking may lead to compression under some circumstances, but their data come from an idiosyncratic cued serial recall task. Here we used the two tasks that have been most widely used to study chunking: 2AFC recognition and immediate serial recall. We also varied the size of the chunks.

The experiments reported here were designed to distinguish between two alternative hypotheses as to how chunking might operate in verbal STM. According to a data compression view, words are recoded into chunks and this should free up space for more words to be stored in memory. This should lead to improved recall of both multiword chunks and singletons. Performance should improve equally for all items in the list regardless of whether they form part of larger chunks. According to a redintegration view, the benefit of chunking should apply to the chunks (pairs or triples) themselves and have or no influence on items not forming part of a larger chunk (singletons).

The results of Experiments 1 and 2, using a recognition task and chunks of two words, strongly favor redintegration. In line with previous studies, overall performance was better for lists containing more pairs. However, contrary to the predictions of a compression hypothesis, the performance on both singletons and pairs was unaffected by the number of pairs in the list, and pairs were remembered better than singletons. The improvement in performance on lists with more pairs was entirely attributable to the fact that they contained more pairs, each of which was always recalled equally well regardless of the composition of the list. There was no indication that the words in a chunk could be stored in a slot that would normally hold just a single item. Instead, the data are exactly as would be expected on the basis of redintegration. Larger chunks are better recalled because the constraints provided by representations of those chunks in LTM enable those chunks to be more readily decoded from STM. As would be expected from a redintegration account, the benefit of chunking does not extend to words that do not form part of a chunk.

The results from the serial recall task used in Experiment 3 are similar. Recall of pairs remains constant regardless of the number of chunks. However, recall of single items does improve when the lists contain more chunks. This occurs most strongly when a strict serial recall scoring procedure is adopted. A possible explanation for this result is that when lists contain more chunks, this places increasing constraints on the number of locations where a single item might appear. It seems that the improvement in recall of singles with increasing number of chunks is entirely attributable to the improvement in recall of the item in Position 5. Note that if compression had been operating, performance on pairs should also have improved as the number of pairs increased, but it did not. These first three experiments make the important point that when recall is found to benefit from the presence of familiar chunks this should not be taken as evidence that the input has been recoded into compressed representations of those chunks in STM.

In Experiments 4, 5, and 6 we used three-word chunks. Given that there must be some cost to chunking, we hypothesized that compression might emerge only with larger chunks. Chunks in the input need to be recognized as such and then recoded into a different compressed representation. At recall, the compressed representation must be unpacked to retrieve the individual items in the chunk. If any of these processes compete for resources or time with memory, then the cost may outweigh the benefits of recoding. In all three of these experiments with three-item chunks, recall of single items improved as the number of triples in the lists increased. In Experiments 5 and 6, using serial recall, performance on triples also improved when there were more triples in the list. That is, when we increased the size of the chunks from two to three items, we saw the emergence of a pattern of data that we take to be a signature of compression. These findings using 2AFC and a standard serial recall task indicate that the data from Thalmann et al. (2019) are not simply due to their use of an unusual task. Of course, it is quite possible that redintegration continued to play some role even when we can be fairly sure that there is compression too. If recall can benefit from redintegration, then it should be used all of the time.

Our initial discussion of chunking models implicitly assumed that the presence of chunks should always make more storage capacity available for all other items in the list. Simple models such as those considered by Cowan et al. (2012) also predict that recall of chunks should be equivalent regardless of the number of words in the chunk, because each chunk must occupy a single slot. However, the most consistent finding across all six experiments was that the larger chunks were recalled better than singletons. This result is consistent with redintegration. However, it can also be explained by compression when we take account of the fact that verbal material must be presented and processed sequentially. If all processing is sequential, then the presence of multiword chunks can only benefit recall of singletons when those chunks appear before the singletons in the list. The serial position data suggest that list items are indeed encoded in strict serial order and are not recoded when later items arrive.

We simulated our serial recall data with a model incorporating the simplest possible account of chunking; as each chunk arrives it is stored exactly as though it were a single item. The simulations gave a good fit to the data. They also explain why performance on chunks should be better than singletons; chunks should be less susceptible to decay or interference during recall. As with all computational models of verbal STM, our simulations do not assume that there is a hard chunk-based capacity limit. Simple fixed-slot chunking models are likely to be more applicable to visual stimuli where all of the items to be remembered can be presented simultaneously.

## Figures and Tables

**Table 1 tbl1:** Design Features of the Six Experiments

Experiment	Task	Chunk size	Chunk presentation	List length	Extra training	Articulatory suppression
1	2AFC	Pair	Chunk	7	No	Yes
2	2AFC	Pair	Chunk	7	Yes	Yes
3	ISR	Pair	Single	7	No	Yes
4	2AFC	Triple	Chunk	7	Yes	Yes
5	ISR	Triple	Single	6	Yes	Yes
6	ISR	Triple	Single	7	Yes	No
*Note*. 2AFC = 2-alternative forced choice; ISR = immediate serial recall. Chunk presentation indicates whether items were presented singly or as chunks. See text for details of extra training.						

**Table 2 tbl2:** Composition of Lists in Experiments 1–3

List condition	Number of words	Number of chunks
1222	7	4
11122	7	5
111112	7	6
1111111	7	7
*Note*. Conditions indicate composition of the list and not the order in which chunks of a give size appear. For example, 1222 refers to a list with 1 singleton and 3 pairs, irrespective of where in the list the singleton appears.		

**Table 3 tbl3:** Composition of Lists in Experiments 4 and 6

List condition	Number of words	Number of chunks
133	7	3
11113	7	5
111111	7	7

**Table 4 tbl4:** Composition of Lists in Experiments 5

List condition	Number of words	Number of chunks
33	6	2
1113	6	4
111111	6	6

**Table 5 tbl5:** Accuracy of Recall of the Three Items in a Triple as a Function of Starting Position of the Triple in Experiment 5

Starting position	First	Second	Third
1	.90	.90	.90
2	.87	.87	.86
3	.77	.81	.78
4	.84	.85	.86

**Table 6 tbl6:** Accuracy of Recall of the Three Items in a Triple as a Function of Starting Position of the Triple in Experiment 6

Starting position	First	Second	Third
1	.94	.93	.93
2	.98	.98	.96
3	.98	.96	.96
4	.92	.92	.84

## Footnotes

1 The rather more complicated Adaptive Huffman code can update the code as it processes a message but is less efficient.

2 Note that this latter prediction may not hold for all possible models of compression.

3 Simulations were written in Python.

4 The same is true here. k for Experiments 1, 2, and 4, from most chunks (all singletons) to fewest (most pairs/triples): E1: 2.5, 3.1, 2.7, and 2.3. E2: 4.6, 3.9, 3.5, and 2.8. E4: 3.8, 3.2, and 2.4.

## Stimuli Used in Experiments

**Table:**

Chunks used in each experiment			
Word pairs used in Experiments 1, 2, and 3			
door knob	game play	pig tail	cave man
finger nail	horse fly	gold fish	goose down
house work	sales woman	bee sting	ice cold
brief case	bus pass	jazz band	school dinner
hack saw	head light	lady bird	table cloth
red rose	monkey business	sail boat	egg shell
shoe lace	radio show	oil can	post box
step mother	mouse trap	winter coat	fruit cake
green tea	desk lamp	shopping list	pie crust
brick building	swim pool	dairy farm	grass seed
river flow	bread bake	coal mine	rain cloud
Word triplets used in Experiment 4			
brass door knob	fair game play	curly pig tail	
extinct cave man	broken finger nail	horse fly bite	
gold fish bowl	goose down quilt	house work done	
busy sales woman	bee sting sore	ice cold beer	
heavy brief case	student bus pass	loud jazz band	
healthy school dinner	sharp hack saw	bright head light	
tiny lady bird	clean table cloth	red rose smell	
cheeky monkey business	moored sail boat	crack egg shell	
black shoe lace	morning radio show	rusty oil can	
sweet green tea	wicked step mother	mouse trap cheese	
warm winter coat	iced fruit cake	parcel post man	
folding desk lamp	long shopping list	cherry pie crust	
Word triplets used in Experiments 5 & 6			
brass door knob	fair game play	curly pig tail	
extinct cave man	broken finger nail	horse fly bite	
gold fish bowl	goose down quilt	house work done	
busy sales woman	bee sting sore	ice cold beer	
heavy brief case	student bus pass	loud jazz band	
healthy school dinner	sharp hack saw	bright head light	
tiny lady bird	clean table cloth	red rose smell	
cheeky monkey business	moored sail boat	crack egg shell	
black shoe lace	morning radio show	rusty oil can	
parcel post service	wicked step mother	mouse trap cheese	
warm winter coat	iced fruit cake	sweet green tea	
folding desk lamp	long shopping list	cherry pie crust	
brown brick building	swim pool full	dairy farm cows	
grass seed grown	fast river flow	hot bread bake	
deep coal mine	dark rain cloud	milk bottle top	

## References

[c45] Baddeley A. D., & Hitch G. (1974). Working memory In Bower G. A. (Ed.), Psychology of learning and motivation (Vol. 8, pp. 47–89). New York, NY: Academic Press.

[c1] Botvinick M. M. (2005). Effects of domain-specific knowledge on memory for serial order. Cognition, 97, 135–151. 10.1016/j.cognition.2004.09.007 16226560

[c2] Botvinick M., & Bylsma L. M. (2005). Regularization in short-term memory for serial order. Journal of Experimental Psychology: Learning, Memory, and Cognition, 31, 351–358. 10.1037/0278-7393.31.2.351 15755251

[c3] Bradmetz J., & Mathy F. (2008). Response times seen as decompression times in Boolean concept use. Psychological Research, 72, 211–234. 10.1007/s00426-006-0098-7 17093950

[c4] Brady T. F., Konkle T., & Alvarez G. A. (2009). Compression in visual working memory: Using statistical regularities to form more efficient memory representations. Journal of Experimental Psychology: General, 138, 487–502. 10.1037/a0016797 19883132

[c5] Brown G. D. A., & Hulme C. (1995). Modeling item length effects in memory span: No rehearsal needed? Journal of Memory and Language, 34, 594–621. 10.1006/jmla.1995.1027

[c6] Chekaf M., Cowan N., & Mathy F. (2016). Chunk formation in immediate memory and how it relates to data compression. Cognition, 155, 96–107. 10.1016/j.cognition.2016.05.024 27367593 PMC4983232

[c7] Chen Z., & Cowan N. (2005). Chunk limits and length limits in immediate recall: A reconciliation. Journal of Experimental Psychology: Learning, Memory, and Cognition, 31, 1235–1249. 10.1037/0278-7393.31.6.1235 PMC2673719 16393043

[c8] Chen Z., & Cowan N. (2009). Core verbal working-memory capacity: The limit in words retained without covert articulation. Quarterly Journal of Experimental Psychology, 62, 1420–1429. 10.1080/17470210802453977 PMC2693080 19048451

[c46] Conrad R. (1965). Order error in immediate recall of sequences. Journal of Verbal Learning and Verbal Behavior, 4, 161–169.

[c9] Cowan N. (2001). The magical number 4 in short-term memory: A reconsideration of mental storage capacity. Behavioral and Brain Sciences, 24, 87–114. 10.1017/S0140525X01003922 11515286

[c10] Cowan N., & Chen Z. (2008). How chunks form in long-term memory and affect short-term memory limits In Page M. & Thorn A. (Eds.), Interactions between short-term and long-term memory in the verbal domain (pp. 86–107). Hove, UK: Psychology Press.

[c11] Cowan N., Chen Z., & Rouder J. N. (2004). Constant capacity in an immediate serial-recall task: A logical sequel to Miller (1956). Psychological Science, 15, 634–640. 10.1111/j.0956-7976.2004.00732.x 15327636

[c12] Cowan N., Rouder J. N., Blume C. L., & Saults J. S. (2012). Models of verbal working memory capacity: What does it take to make them work? Psychological Review, 119, 480–499. 10.1037/a0027791 22486726 PMC3618891

[c13] Farrell S., & Lewandowsky S. (2002). An endogenous distributed model of ordering in serial recall. Psychonomic Bulletin & Review, 9, 59–79. 10.3758/BF03196257 12026954

[c14] Forster K. I., & Forster J. C. (2003). DMDX: A windows display program with millisecond accuracy. Behavior Research Methods, Instruments & Computers, 35, 116–124. 10.3758/BF03195503 12723786

[c15] Glanzer M., & Fleishman J. (1967). The effect of encoding training on perceptual recall. Perception & Psychophysics, 2, 561–564. 10.3758/BF03210268

[c16] Huang L., & Awh E. (2018). Chunking in working memory via content-free labels. Scientific Reports, 8, 23 10.1038/s41598-017-18157-5 29311568 PMC5758528

[c17] Hulme C., Maughan S., & Brown G. D. (1991). Memory for familiar and unfamiliar words: Evidence for a long-term memory contribution to short-term memory span. Journal of Memory and Language, 30, 685–701. 10.1016/0749-596X(91)90032-F

[c18] Hulme C., Roodenrys S., Brown G., & Mercer R. (1995). The role of long-term-memory mechanisms in memory span. British Journal of Psychology, 86, 527–536. 10.1111/j.2044-8295.1995.tb02570.x

[c19] Hulme C., Roodenrys S., Schweickert R., Brown G. D., Martin M., & Stuart G. (1997). Word-frequency effects on short-term memory tasks: Evidence for a redintegration process in immediate serial recall. Journal of Experimental Psychology: Learning, Memory, and Cognition, 23, 1217–1232. 10.1037/0278-7393.23.5.1217 9293631

[c20] Jeffreys H. (1961). Theory of probability (3rd ed.). Oxford, UK: Oxford University Press.

[c21] Jones T., & Farrell S. (2018). Does syntax bias serial order reconstruction of verbal short-term memory? Journal of Memory and Language, 100, 98–122. 10.1016/j.jml.2018.02.001

[c22] Kalm K., Davis M. H., & Norris D. (2013). Individual sequence representations in the medial temporal lobe. Journal of Cognitive Neuroscience, 25, 1111–1121. 10.1162/jocn_a_00378 23448522

[c23] Kalm K., & Norris D. (2016). Recall is not necessary for verbal sequence learning. Memory & Cognition, 44, 104–113. 10.3758/s13421-015-0544-0 26289546 PMC4722071

[c24] Kass R. E., & Raftery A. E. (1995). Bayes factors. Journal of the American Statistical Association, 90, 773–795. 10.1080/01621459.1995.10476572

[c25] Kleinberg J., & Kaufman H. (1971). Constancy in short-term memory: Bits and chunks. Journal of Experimental Psychology, 90, 326–333. 10.1037/h0031563 5134339

[c26] Levenshtein V. I. (1966). Binary codes capable of correcting deletions, insertions, and reversals. Soviet Physics Doklady, 10, 707–710.

[c27] Lewandowsky S., & Farrell S. (2000). A redintegration account of the effects of speech rate, lexicality, and word frequency in immediate serial recall. Psychological Research, 63, 163–173. 10.1007/PL00008175 10946590

[c28] Lewandowsky S., & Farrell S. (2008). Short-term memory: New data and a model. Psychology of Learning and Motivation, 49, 1–48. 10.1016/S0079-7421(08)00001-7

[c29] Luck S. J., & Vogel E. K. (1997). The capacity of visual working memory for features and conjunctions. Nature, 390, 279–281. 10.1038/36846 9384378

[c30] Mathy F., & Feldman J. (2012). What’s magic about magic numbers? Chunking and data compression in short-term memory. Cognition, 122, 346–362. 10.1016/j.cognition.2011.11.003 22176752

[c31] Mathy F., & Varré J.-S. (2013). Retention-error patterns in complex alphanumeric serial-recall tasks. Memory, 21, 945–968. 10.1080/09658211.2013.769607 23485108

[c32] Miller G. A. (1956). The magical number seven plus or minus two: Some limits on our capacity for processing information. Psychological Review, 63, 81–97. 10.1037/h0043158 13310704

[c33] Norris D., & Kalm K. (2019). What’s in a chunk? Chunking and data compression in verbal short-term memory [preprint]. PsyArXiv 10.31234/osf.io/sm736 33360054

[c34] Page M. P., & Norris D. (1998). The primacy model: A new model of immediate serial recall. Psychological Review, 105, 761–781. 10.1037/0033-295X.105.4.761-781 9830378

[c35] Poirier M., & Saint-Aubin J. (1996). Immediate serial recall, word frequency, item identity and item position. Canadian Journal of Experimental Psychology/Revue canadienne de psychologie expérimentale, 50, 408–412. 10.1037/1196-1961.50.4.408 9025332

[c36] Pollack I., & Johnson L. B. (1965). Memory-span with efficient coding procedures. The American Journal of Psychology, 78, 609–614. 10.2307/1420923 5839929

[c37] Portrat S., Guida A., Phénix T., & Lemaire B. (2016). Promoting the experimental dialogue between working memory and chunking: Behavioral data and simulation. Memory & Cognition, 44, 420–434. 10.3758/s13421-015-0572-9 26597851

[c38] Powell M. J. D. (1964). An efficient method for finding the minimum of a function of several variables without calculating derivatives. The Computer Journal, 7, 155–162. 10.1093/comjnl/7.2.155

[c39] Roodenrys S., & Miller L. M. (2008). A constrained Rasch model of trace redintegration in serial recall. Memory & Cognition, 36, 578–587. 10.3758/MC.36.3.578 18491497

[c40] Schneider W. E., & Zuccolotto A. (2002). E-Prime user’s guide. Pittsburgh, PA: Psychology Software Tools, Inc.

[c41] Schweickert R. (1993). A multinomial processing tree model for degradation and redintegration in immediate recall. Memory & Cognition, 21, 168–175. 10.3758/BF03202729 8469125

[c42] Simon H. A. (1974). How big is a chunk?: By combining data from several experiments, a basic human memory unit can be identified and measured. Science, 183, 482–488. 10.1126/science.183.4124.482 17773029

[c43] Thalmann M., Souza A., & Oberauer K. (2019). How does chunking help working memory? Journal of Experimental Psychology: Learning, Memory, and Cognition, 45, 37–55. 10.1037/xlm0000578 29698045

[c44] Thorn A. S., Gathercole S. E., & Frankish C. R. (2002). Language familiarity effects in short-term memory: The role of output delay and long-term knowledge. The Quarterly Journal of Experimental Psychology A: Human Experimental Psychology, 55, 1363–1383. 10.1080/02724980244000198 12420999

